# M-NGO/UiO-66 nanocomposite: a green and efficient catalyst for the synthesis of 1,8-dioxodecahydroacridine derivatives

**DOI:** 10.1039/d6ra03005j

**Published:** 2026-07-17

**Authors:** Atefeh Zeinali, Leila Moradi

**Affiliations:** a Department of Organic Chemistry, Faculty of Chemistry, University of Kashan P. O. Box 8731753153 Kashan I.R. Iran l_moradi@kashanu.ac.ir

## Abstract

In this study, a novel magnetic nanocomposite (M-NGO/UiO-66) was successfully synthesized. This nanocomposite was utilized as a green, heterogeneous, and recoverable catalyst in a one-pot pseudo-four-component reaction for the preparation of 1,8-dioxodecahydroacridine derivatives. The structure and physicochemical properties of the resulting nanocomposite were thoroughly characterized and confirmed using FT-IR, XRD, SEM, EDS, elemental mapping, BET, and VSM techniques. The catalytic performance of M-NGO/UiO-66 was evaluated under optimized reaction conditions, including 0.015 g of catalyst in ethanol at 80 °C, affording the target products within 8–25 min and in high yields ranging from 83% to 95%. The developed protocol demonstrates several significant advantages, such as operational simplicity, excellent catalytic activity, high atom economy, favorable reaction mass efficiency, utilization of a green solvent, and convenient magnetic recovery of the catalyst, making it an attractive and sustainable approach for the synthesis of acridine derivatives.

## Abstract 2

A novel magnetic nanocomposite (M-NGO/UiO-66) was successfully prepared and utilized for the green synthesis of 1,8-dioxodecahydroacridine derivatives.

## Graphical abstract

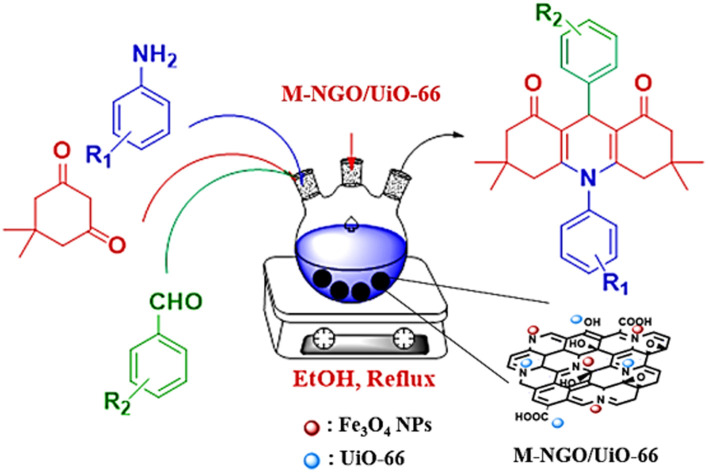

## Introduction

Sustainable chemistry, often referred to as green chemistry, has emerged as a critical approach for designing chemical processes that minimize environmental impact.^1,2^ Guided by the 12 principles outlined by Anastas and Warner, this field prioritizes waste prevention, atom economy, and the use of safer reagents and solvents from the very beginning of process design.^3–7^ A powerful manifestation of these principles is the application of multicomponent reactions (MCRs), in which three or more reactants combine in a single vessel to produce a complex molecule.^8–10^ MCRs significantly reduce the number of purification steps, lower energy consumption, and minimize waste generation, making them highly attractive for both drug discovery and materials science.^11–17^

Among the various classes of compounds accessible through MCRs, nitrogen-containing heterocycles occupy a privileged position due to their widespread presence in bioactive molecules, pharmaceuticals, natural products, and functional materials.^18–25^ In particular, 1,8-dioxodecahydroacridines have attracted considerable attention because of their promising biological activities, including antimalarial, anticancer, and antimicrobial properties.^26–28^ These compounds are commonly synthesized *via* a pseudo-four-component reaction involving dimedone (2 mmol), aromatic aldehydes, and aniline derivatives. Although numerous catalytic methods have been developed for their preparation, many of them still suffer from drawbacks such as long reaction times, harsh conditions, difficult catalyst recovery, and the use of toxic solvents. Therefore, the development of efficient, sustainable, and recyclable heterogeneous catalysts for this transformation remains an important goal.^29–41^

Heterogeneous catalysis offers a practical solution by providing easy separation, reusability, and reduced product contamination.^42,43^ In organic transformations, heterogeneous catalysts can minimize side products and provide precise control over selectivity, often through the incorporation of multiple active sites on high-surface-area supports.^44–47^ In recent years, graphene oxide (GO) and its nitrogen-doped derivative (NGO) have been extensively explored as catalyst supports owing to their high surface area, rich oxygen- and nitrogen-containing functional groups, excellent mechanical stability, and tunable electronic properties.^48–55^ Nitrogen doping further increases the density of active sites and improves the catalytic performance of GO in various organic transformations.^51,52^ However, NGO-based catalysts still face limitations such as relatively low surface area compared to highly porous materials, difficult recovery from reaction mixtures, and moderate stability under prolonged catalytic cycles.^56^

To address these limitations, Metal–Organic Frameworks (MOFs) have emerged as promising materials for hybridization with carbon-based supports.^57^ MOFs are crystalline porous materials constructed from metal clusters and organic linkers, offering exceptionally high surface areas, tunable pore sizes, and abundant active sites.^58^ Among them, UiO-66, a zirconium-based framework built from Zr_6_O_4_(OH)_4_ clusters and terephthalic acid linkers, stands out due to its outstanding thermal and chemical stability, high porosity, and defect sites that can be engineered for enhanced catalytic activity.^59–64^

In addition to high surface area and active site density, facile recovery and reusability are essential features of advanced heterogeneous catalysts.^65^ Incorporating superparamagnetic Fe_3_O_4_ nanoparticles into the nanocomposite design imparts excellent magnetic separability. This allows rapid recovery of the catalyst from the reaction mixture using an external magnet, without the need for filtration or centrifugation, thereby reducing solvent consumption and operational time while perfectly aligning with green chemistry principles.^66–69^

**Scheme 1:**
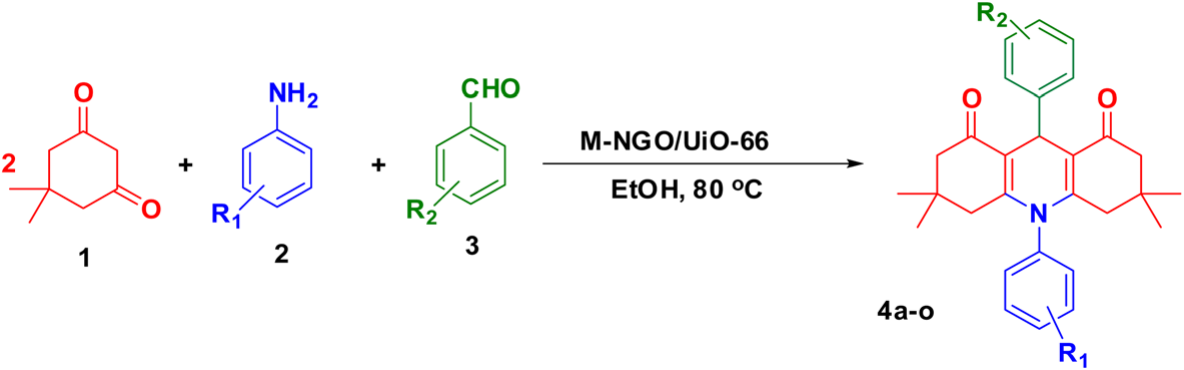
Preparation of 1,8-dioxodecahydroacridine derivatives using M-NGO/UiO-66.

In the present work, we report the design and synthesis of a novel magnetically separable M-NGO/UiO-66 nanocomposite as a highly efficient heterogeneous catalyst for the one-pot, pseudo-four-component synthesis of 1,8-dioxodecahydroacridine derivatives in ethanol at 80 °C (Scheme 1). This hybrid system successfully integrates the catalytic benefits of nitrogen-doped graphene oxide (NGO) with the high porosity and stability of UiO-66, while the incorporation of Fe_3_O_4_ nanoparticles ensures facile magnetic recovery. Although N-GO and UiO-66 have been used individually in various multicomponent reactions,^70–73^ based on our studies, this is the first report combining them into a magnetic hybrid catalyst specifically applied to the synthesis of 1,8-dioxodecahydroacridines. Consequently, the developed M-NGO/UiO-66 nanocomposite offers distinct advantages over previously reported catalysts for this transformation, including shorter reaction times, higher yields, effortless magnetic separation, excellent recyclability, and the use of ethanol as a green solvent.

## Experimental section

### Chemicals and instrumentation

All chemicals and solvents were procured from Merck & Aldrich and Fluka companies. The progress of reactions was monitored using silica gel TLC plates (Merck, FG254). Nuclear magnetic resonance (NMR) spectra, including ^1^H NMR (400 MHz) and ^13^C NMR (100 MHz), were recorded on a Bruker DRX-400 spectrometer with DMSO-*d*_6_ and CDCl_3_ as solvent and tetramethylsilane as the internal standard. Fourier-transform infrared (FT-IR) spectra were obtained using a PerkinElmer 781 spectrophotometer with samples prepared as KBr pellets. The morphology of the nanocatalyst was analyzed *via* field-emission scanning electron microscopy by model of TESCAN MIRA3. Crystallographic properties were investigated through X-ray diffraction (XRD) using a Bruker D8 Advance diffractometer with Cu Kα radiation (*λ* = 1.5406 Å). Surface area and pore structure characteristics were determined from nitrogen adsorption–desorption isotherms measured on a Micromeritics TriStar 3000 analyzer, with specific surface areas calculated using the Brunauer–Emmett–Teller (BET) method at 77 K *via* a BELSORP Mini II instrument. Magnetic properties were evaluated using a vibrating sample magnetometer by model of Daghigh Meghnatis Kashan Co. Melting points were measured in sealed capillary tubes with a Thermo Scientific electrothermal melting point apparatus Model 9100 BZ, UK.

## Catalyst preparation

### Preparation of graphene oxide (GO)

Graphene oxide was synthesized using a modified Hummers' method.^74^ A mixture of 2 g graphite powder, 1 g sodium nitrate, and 6 g potassium permanganate was added to 60 mL of 98% sulfuric acid. The mixture was stirred in an ice bath for 90 minutes, followed by stirring at 40 °C for 2 hours. Subsequently, 150 mL of deionized water was added, and the temperature was raised to 98 °C with stirring for 30 minutes. After cooling to 60 °C, 10 mL of 30% hydrogen peroxide was added, and the mixture was stirred for an additional 2 hours. Then, the resulting product was filtered, washed with 5% hydrochloric acid and deionized water until a neutral pH was achieved. The obtained graphite oxide was then dispersed in deionized water and stirred overnight to achieve a homogeneous suspension. Subsequently, the suspension was ultrasonicated for 30–60 minutes to exfoliate the oxidized graphite layers into graphene oxide (GO) nanosheets. The resulting dispersion was centrifuged at 3000 rpm for 30 minutes to remove unexfoliated graphite particles and large aggregates. Finally, the supernatant containing well-exfoliated graphene oxide (GO) nanosheets was collected and dried at 60 °C for further characterization and application.

### Preparation of nitrogen-doped graphene oxide (N-GO)

To prepare nitrogen-doped graphene oxide, 0.2 g of graphene oxide was dispersed in 90 mL of deionized water, followed by the addition of 20 g of urea. The mixture was sonicated for 1 hour and then transferred to an autoclave and heated at 180 °C for 12 hours. The resulting black solid was separated by centrifugation, washed multiple times with deionized water, and dried in an oven at 80 °C.^75^

### Preparation of magnetic NGO nanocomposite (M-NGO)

0.1 g of graphene oxide powder was dispersed in 150 mL of deionized water *via* sonication for 30 minutes. Subsequently, 1.6 g (5 mmol) of FeCl_3_·6H_2_O and 0.6 g (3 mmol) of FeCl_2_·4H_2_O were added to the mixture and then, 25 mL of 30% ammonium hydroxide was added dropwise under nitrogen atmosphere with continuous stirring. The resulting black solution was stirred at 80 °C for 1 hour. Finally, the obtained precipitate was separated using a magnet, washed multiple times with deionized water and ethanol, and dried in an oven at 80 °C.

### Deposition of UiO-66 to M-NGO surfaces (M-NGO/UiO-66)

0.3 g of the prepared M-NGO was dispersed in 80 mL of DMF *via* sonication for 10 minutes. Then, 0.12 g (0.5 mmol) of ZrCl_4_ and 0.08 g (0.5 mmol) of terephthalic acid (TPA) was added, and followed by stirring at room temperature until homogeneity was reached. Subsequently the resulting solution was transferred to an autoclave and heated at 120 °C for 24 hours. The obtained precipitate was separated by centrifugation, washed 3 times with deionized water and ethanol and dried in an oven.^76,77^

### General procedure for the synthesis of 1,8-dioxodecahydroacridine derivatives

A reaction mixture containing of an aniline derivative (1 mmol), dimedone (1 mmol) and M-NGO/UiO-66 nanocatalyst (0.015 g) in 3 mL of ethanol was prepared and stirred at 80 °C for 5 minutes (to produce the first intermediate). Subsequently, 1 mmol of aldehyde and 1 mmol of dimedone were added, and the mixture was stirred until the reaction completion (as confirmed by TLC). Upon completion, the magnetic nanocatalyst was separated from the reaction mixture using an external magnetic field and the product was isolated *via* filtration and purified by recrystallization from ethanol. All of resulting compounds were characterized through spectroscopic methods. All products were characterized by spectroscopic methods; the corresponding spectra and complete data are available in the SI.

## Results and discussion

### Characterization of M-NGO/UiO-66

**Scheme 2:**
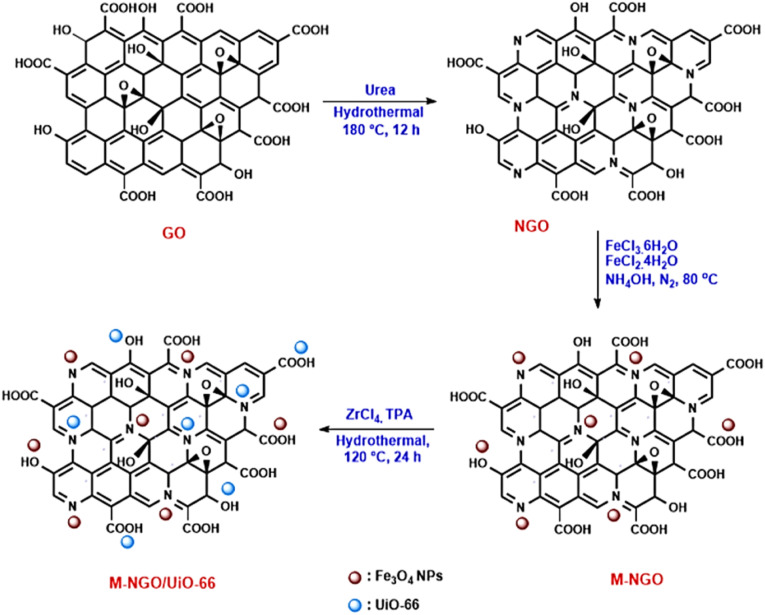
The synthesis steps of the M-NGO/UiO-66 nanocomposite.

To prepare the M-NGO/UiO-66 nanocomposite, graphene oxide was first subjected to hydrothermal treatment with urea, resulting in nitrogen-doped graphene oxide (NGO). Subsequently, Fe_3_O_4_ magnetic nanoparticles were anchored onto the NGO substrate to impart magnetic properties. Finally, to deposit the metal–organic framework (UiO-66 MOF) on M-NGO nanosheets surfaces, the UiO-66 structure was synthesized (*in situ*) by combining ZrCl_4_ as metal precursor and terephthalic acid (TPA) as organic ligand. The entire process was conducted under controlled hydrothermal conditions, and after washing and drying, the final product was utilized as M-NGO/UiO-66 nanocomposite (Scheme 2).

### FT-IR analysis

**Fig. 1:**
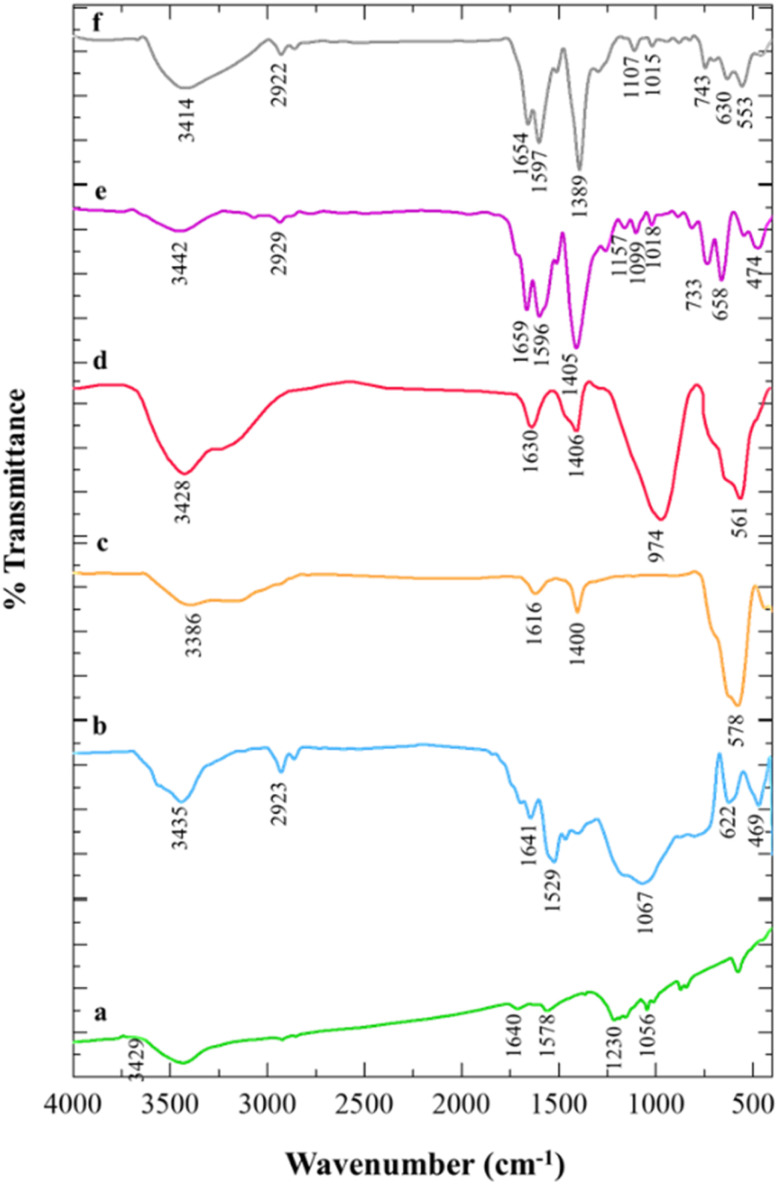
FT-IR spectra of GO (a), N-GO (b), Fe_3_O_4_ nanoparticles (c), M-NGO (d), UiO-66 (e) and M-NGO/UiO-66 (f).

To investigate the functional groups and confirm the successful synthesis of M-NGO/UiO-66, FT-IR spectra of GO, NGO, Fe_3_O_4_, M-NGO, UiO-66, and M-NGO/UiO-66 were recorded (Fig. 1a–f). The FT-IR spectrum of GO (Fig. 1a) shows a broad O–H stretching band at 3429 cm^−1^ (hydroxyl groups and adsorbed water), C=O stretching at 1640 cm^−1^, aromatic C=C at 1578 cm^−1^, and C–O vibrations at 1230 and 1056 cm^−1^. In the NGO spectrum (Fig. 1b), characteristic peaks appear at 3435 cm^−1^ (O–H and N–H), 1641 cm^−1^ (C=O), 1067 cm^−1^ (C–N and C–O), and 2923 cm^−1^ (aliphatic C–H), confirming successful nitrogen doping into the GO structure.^78^ The Fe_3_O_4_ spectrum (Fig. 1c) exhibits a strong Fe–O stretching band at 578 cm^−1^ (tetrahedral site), along with O–H stretching and bending vibrations at 3386 and 1616 cm^−1^, consistent with the pure magnetite phase.^79^ For M-NGO (Fig. 1d), the spectrum displays the Fe–O band at 561 cm^−1^ in addition to the characteristic NGO peaks (1630 and 1406 cm^−1^). Slight shifts of these bands indicate interactions between Fe_3_O_4_ nanoparticles and NGO nanosheets. The UiO-66 spectrum (Fig. 1e) shows asymmetric carboxylate stretching at 1596 cm^−1^, adsorbed water bending at 1659 cm^−1^, aromatic C=C at 1405 cm^−1^, C–O/C–N vibrations at 1157, 1099, and 1018 cm^−1^, and Zr–O/Zr–OC bands at 733, 658, and 474 cm^−1^, confirming the successful formation of the MOF framework.^80^ In the final M-NGO/UiO-66 spectrum (Fig. 1f), characteristic bands from all components are observed: carboxylate vibrations at 1654 and 1597 cm^−1^ (UiO-66), Fe–O at 553 cm^−1^, Zr–O at 630 cm^−1^, C–O/C–N bands at 1389, 1107, and 1015 cm^−1^, and aromatic ring vibration at 743 cm^−1^. The co-existence of these peaks with slight shifts relative to the individual components confirms the successful hybridization and effective interactions between M-NGO and UiO-66.

**Fig. 2:**
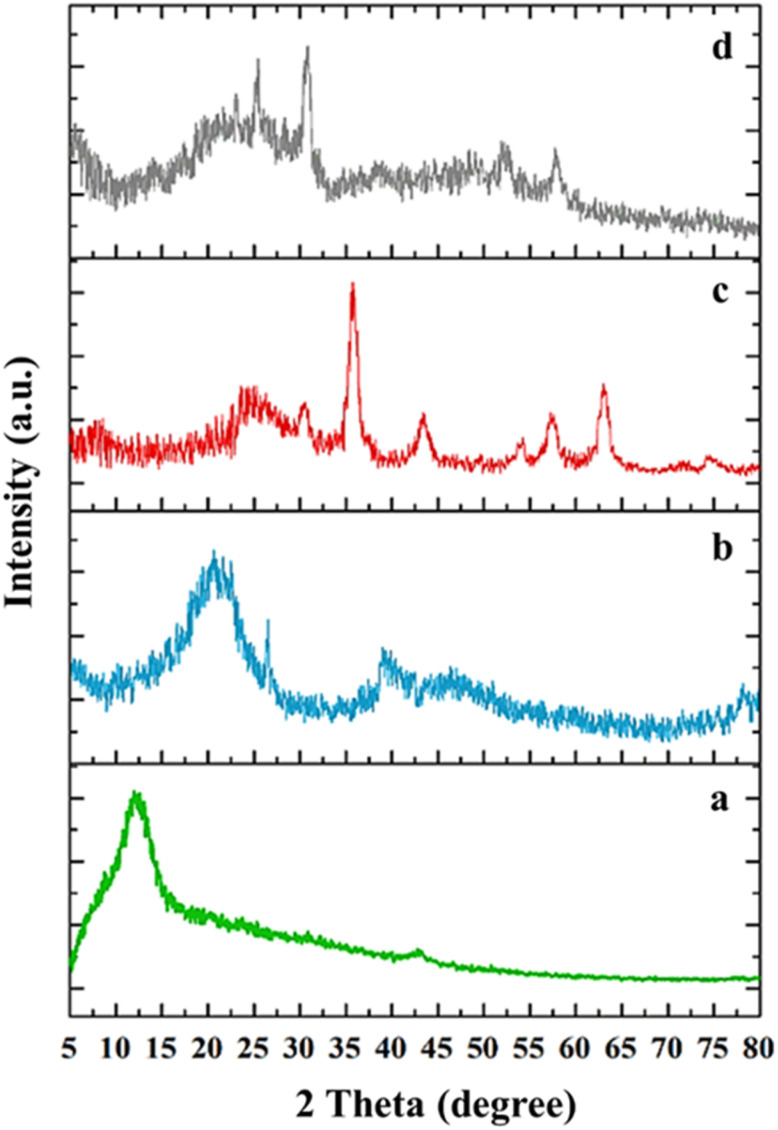
X-ray diffraction pattern of GO (a), N-GO (b), M-NGO (c) and M-NGO/UiO-66 (d).

The XRD patterns of graphene oxide (GO), nitrogen-doped graphene oxide (N-GO), M-NGO nanocomposite, and M-NGO/UiO-66 were recorded using Cu Kα radiation in the 2*θ* range of 5–80° (Fig. 2a–d). The GO pattern (Fig. 2a) shows a strong (001) diffraction peak at 2*θ* ≈ 10.5°, along with a weak broad feature around 2*θ* ≈ 42° related to disordered sp^2^ hybridized regions.^81^ Upon nitrogen doping (Fig. 2b), the (001) peak shifts to 2*θ* ≈ 24°, indicating partial restoration of the graphitic structure with increased interlayer spacing.^80^ The M-NGO pattern (Fig. 2c) retains the N-GO (001) peak at 2*θ* ≈ 26° (slightly shifted due to interaction with Fe_3_O_4_ nanoparticles) and exhibits the characteristic cubic spinel peaks of magnetite (Fe_3_O_4_, JCPDS no. 19-0629) at 2*θ* ≈ 30.2° (220), 35.6° (311), 43.2° (400), 53.5° (422), 57.1° (511), 62.7° (440), and 74.2° (533), confirming successful decoration of N-GO with magnetic nanoparticles.^79^ The final M-NGO/UiO-66 composite (Fig. 2d) displays a superposition of the diffraction peaks from both components. The characteristic Fe_3_O_4_ peaks are preserved, while new peaks at 2*θ* = 7.2°, 7.8°, 17.2°, 22.3°, 25.3°, 30.6°, and 43.1° corresponding to the (111), (200), (004), (044), (006), (117), and (339) planes of UiO-66 (JCPDS no. 0543-83) are clearly observed.^82^ This confirms the successful integration of UiO-66 with M-NGO without disrupting the crystalline structure of either component.

**Fig. 3:**
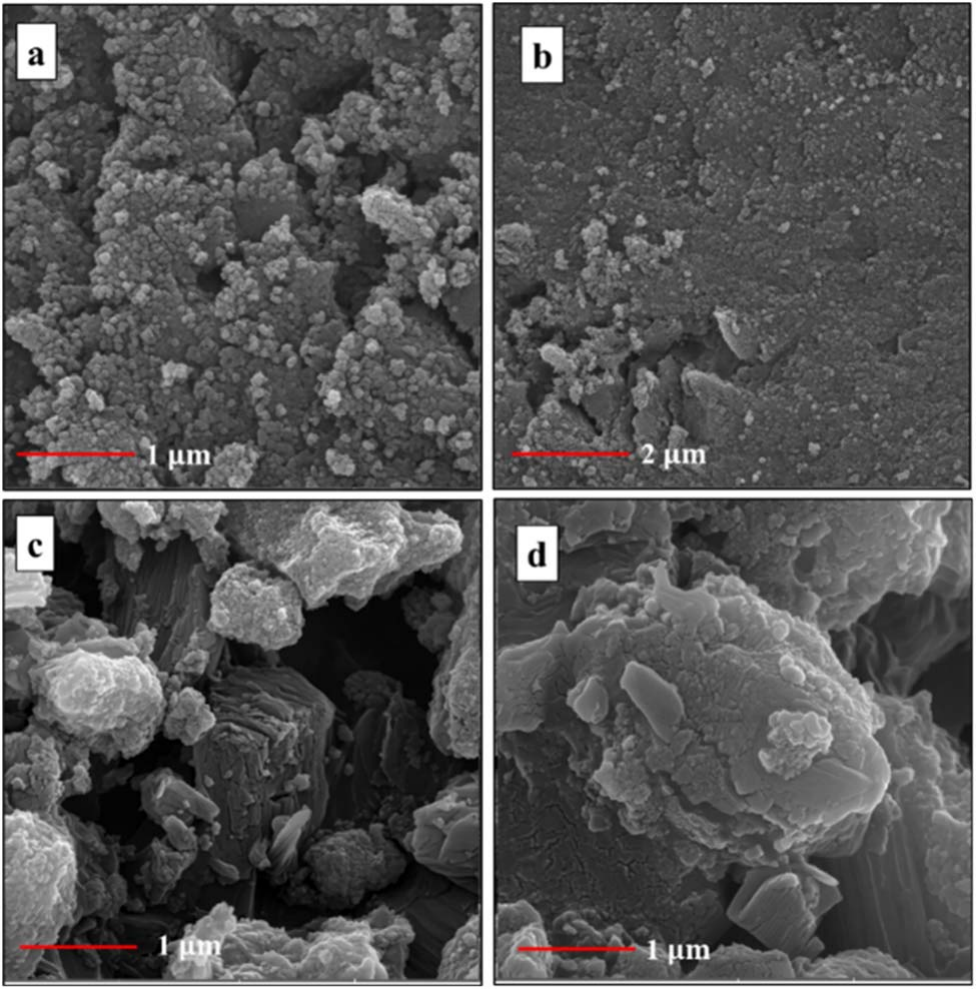
SEM images of M-NGO (a and b) and M-NGO/UiO-66 (c and d).

The SEM images of M-NGO (Fig. 3a and b) and M-NGO/UiO-66 (Fig. 3c and d) provide clear evidence of structural transformations resulting from the combination of M-NGO and UiO-66. As observed in images (a and b), the M-NGO sample consists of uniformly distributed spherical Fe_3_O_4_ nanoparticles on the surfaces of NGO nanolayers. In contrast, images (c) and (d) of the M-NGO/UiO-66 display irregular aggregates in the surfaces of the layered structures, which can be attributed to the formation of UiO-66 MOF crystals on the M-NGO substrate. These morphological changes not only confirm the successful synthesis and formation of the hybrid structure, but also indicate an increase in surface porosity and roughness in the M-NGO/UiO-66 sample. Such characteristics are likely to enhance the catalytic properties of the prepared nanocomposite.

**Fig. 4:**
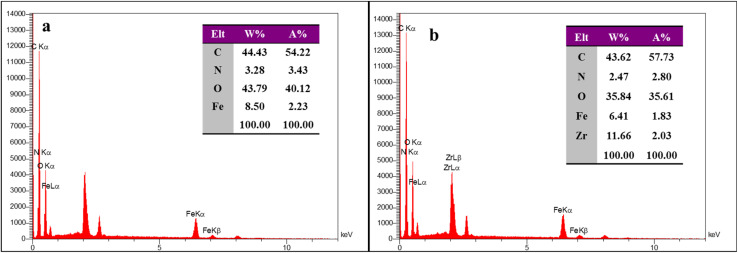
The EDX analysis of the as-prepared of M-NGO (a) and M-NGO/UiO-66 (b).

To thoroughly investigate the elemental composition of the synthesized samples, Energy-Dispersive X-ray Spectroscopy (EDX) analysis was conducted on the M-NGO (Fig. 4a) and M-NGO/UiO-66 (Fig. 4b) samples. In the EDX spectrum of the M-NGO (Fig. 4a), carbon, nitrogen, oxygen, and iron elements are observed. The presence of Fe in this sample indicates the successful loading of Fe_3_O_4_ nanoparticles onto the N-doped graphene oxide structure and the presence of nitrogen confirms the N-doping through the GO nanosheet frameworks. In the M-NGO/UiO-66 sample (Fig. 4b), the presence of Zr element in addition to carbon, nitrogen, oxygen, and iron elements validate the successful surface modification of M-NGO with UiO-66 MOF nanostructures.

**Fig. 5:**
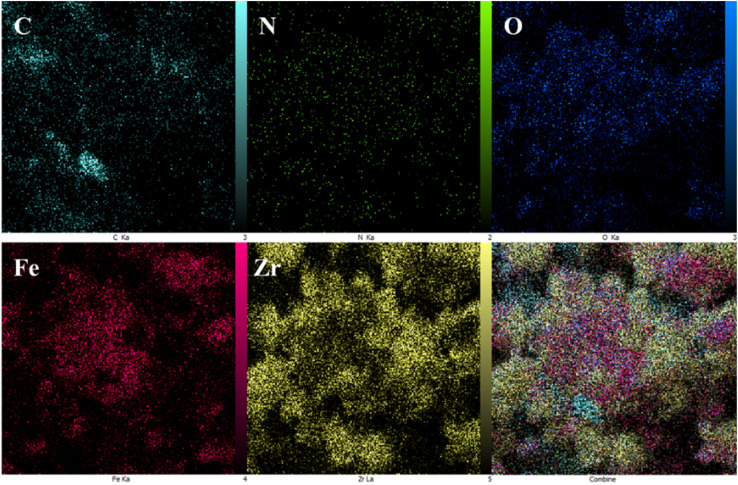
Elemental EDS mapping of M-NGO/UiO-66.

The elemental mapping images of M-NGO/UiO-66 (Fig. 5) demonstrate the uniform and homogeneous distribution of C, N, O, Fe, and Zr throughout the composite. The widespread presence of carbon confirms the structural stability of the N-doped graphene oxide support. The well-distributed nitrogen validates successful N-doping, while the uniform oxygen distribution is attributed to the oxygen-containing functional groups present in both N-GO and UiO-66. Moreover, the non-aggregated dispersion of Fe and Zr indicates effective immobilization and stabilization of Fe_3_O_4_ nanoparticles and UiO-66 crystals on the N-GO sheets. Overall, these results confirm the high homogeneity and structural integrity of the synthesized hybrid catalyst.

**Fig. 6:**
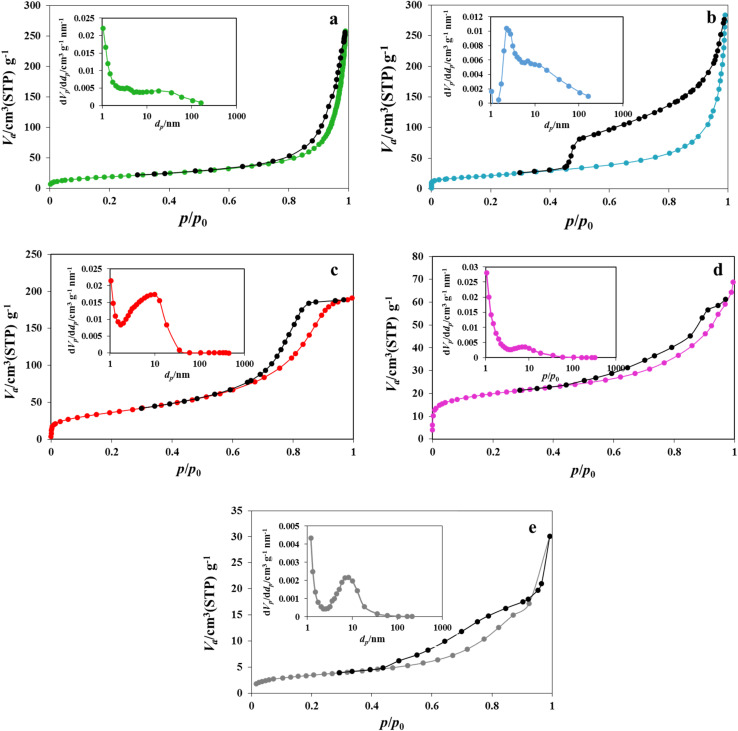
N_2_ adsorption–desorption isotherms and pore size distribution curves of GO (a), NGO (b), M-NGO (c), UiO-66 (d), and M-NGO/UiO-66 (e).

Nitrogen adsorption–desorption measurements were performed to investigate the evolution of the porous structure during the synthesis of GO, N-GO, M-NGO, UiO-66, and M-NGO/UiO-66 (Fig. 6 and Table 1). GO and N-GO exhibited type IV isotherms with H3 hysteresis loops, characteristic of slit-shaped mesopores generated by stacked graphene layers (Fig. 6 a and b). Upon nitrogen doping, the BET surface area increased from 68.41 to 80.57 m^2^ g^−1^, accompanied by slight increases in pore volume and average pore diameter, which can be attributed to the formation of structural defects and suppression of sheet restacking. After deposition of Fe_3_O_4_ nanoparticles, M-NGO displayed a type IV isotherm with an H2(b) hysteresis loop and a significantly higher BET surface area (132.53 m^2^ g^−1^) (Fig. 6c). This increase is mainly attributed to the spacer effect of Fe_3_O_4_ nanoparticles, which prevents aggregation of graphene sheets and generates additional interparticle voids accessible to nitrogen atoms. Simultaneously, the decrease in pore volume and pore diameter suggests partial occupation of larger pores by magnetic nanoparticles. UiO-66 exhibited a type IV isotherm with an H4 hysteresis loop, indicating the coexistence of micro- and narrow mesopores (Fig. 6d). Following the growth of UiO-66 on M-NGO, the resulting M-NGO/UiO-66 nanocomposite showed a type IV isotherm with an H3 hysteresis loop (Fig. 6e). The BET surface area decreased to 12.53 m^2^ g^−1^, while the average pore diameter increased to 8.61 nm. The reduction in surface area is likely associated with partial pore blockage, coverage of accessible graphene surfaces by UiO-66 crystallites, and aggregation during MOF growth. Nevertheless, the preserved mesoporous structure and relatively large pore diameter can still facilitate efficient mass transfer of reactants and products. Moreover, the excellent catalytic performance of M-NGO/UiO-66 demonstrates that the catalytic activity is governed not only by the surface area but also by the presence of abundant accessible active sites, including Lewis acidic Zr^4+^ centers of UiO-66 and nitrogen-containing functionalities of NGO. Therefore, despite the lower BET surface area, the final nanocomposite possesses a suitable porous architecture and highly accessible catalytic sites for the target transformation.

**Table 1:** Textural parameters of the synthesized materials obtained from N_2_ adsorption–desorption measurements

Materials	BET surface area (m^2^ g^−1^)	Total pore volume (cm^3^ g^−1^)	Average pore diameter (nm)
GO	68.412	0.3994	15.878
NGO	80.573	0.427	19.565
M-NGO	132.53	0.2942	7.0159
UiO-66	71.076	0.1009	5.2264
M-NGO/UiO-66	12.528	0.046	8.6104

**Fig. 7:**
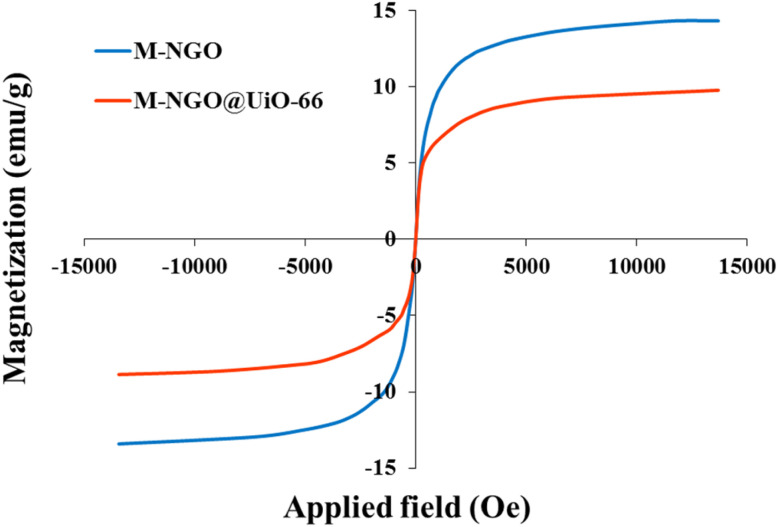
Magnetization curves for M-NGO and M-NGO/UiO-66.

The magnetization curves obtained for the M-NGO and M-NGO/UiO-66 samples confirm the magnetic behavior of both samples (Fig. 7). As evident from the plot, the M-NGO sample exhibits a higher saturation magnetization compared to the M-NGO/UiO-66 sample. The reduction in saturation magnetization is from the incorporation of the UiO-66 MOF as a non-magnetic structure on M-NGO nanosheets.

### Optimization and substrate generality in the synthesis of 1,8-dioxodecahydroacridines catalyzed by M-NGO/UiO-66

To achieve the highest yield and significantly reduce the reaction time in the synthesis of the compound 4h, a comprehensive optimization process was conducted on three key parameters: solvent, catalyst amount and reaction temperature (Table 2). In this study, to evaluate the effect of each parameter on the reaction performance, 2 mmol of dimedone, 1 mmol of 4-chlorobenzaldehyde, and 1 mmol of 4-ethylaniline were employed. Initially, the effect of solvents on yield and reaction rate was investigated. The results indicated that ethanol compare to other applied solvents in the presence of 0.015 g of catalyst, leading to a substantial increase in reaction efficiency (entry 4). Solvents such as chloroform, acetonitrile, H_2_O, and a H_2_O/EtOH mixture resulted the lower yields and higher reaction times (entries 1–3 and 5). The catalyst loading was assessed at the next step. Testing of various amounts of catalyst revealed that 0.015 g of the M-NGO/UiO-66 lead to the highest yield (entry 8) and the reaction without catalyst show trace amount of product after long time (entry 6). Reducing the catalyst to 0.005 g led to decreasing of efficiency and longer time (entry 7), while increasing it to 0.015 g (entry 8) offered no further improvement in performance. In the final stage, the effect of reaction temperature was examined. The findings established the reflux conditions as the ideal temperature, yielding the maximum product efficiency (95%) in the shortest time (6 minutes, entry 4). Lower temperatures, including room temperature, 50 °C, and 60 °C (entries 9–11), resulted the reduced yields and prolonged reaction periods. Based on these evaluations, ethanol as the solvent, 0.01 g of M-NGO/UiO-66 catalyst and reflux conditions are optimal conditions for the synthesis of the compound 4h.

**Table 2:** Optimization of reaction parameters for the synthesis of the compound 4h^a^

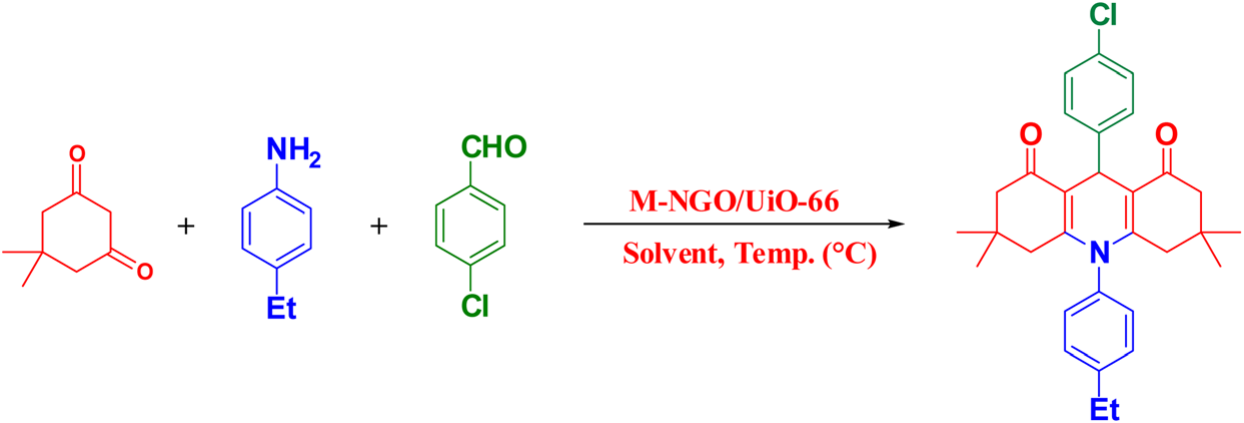					
Entry	Solvent	Catalyst (g)	*T* (°C)	Time (min)	Yield^b^ (%)
1	CHCl_3_	0.01	Reflux	25	30
2	CH_3_CN	0.01	Reflux	18	38
3	H_2_O	0.01	Reflux	20	50
4	EtOH	0.01	Reflux	8	96
5	H_2_O/EtOH	0.01	Reflux	12	85
6	EtOH	—	Reflux	240	10
7	EtOH	0.005	Reflux	20	62
8	EtOH	0.015	Reflux	8	96
9	EtOH	0.01	r.t	120	60
10	EtOH	0.01	50	15	80
11	EtOH	0.01	60	12	85

a Reaction condition: dimedone (2 mmol), 4-chlorobenzaldehyde (1 mmol), 4-ethylaniline (1 mmol), and 3 mL of solvent, in the presence of the M-NGO/UiO-66 composite under thermal conditions.

b Isolated yield.

**Fig. 8:**
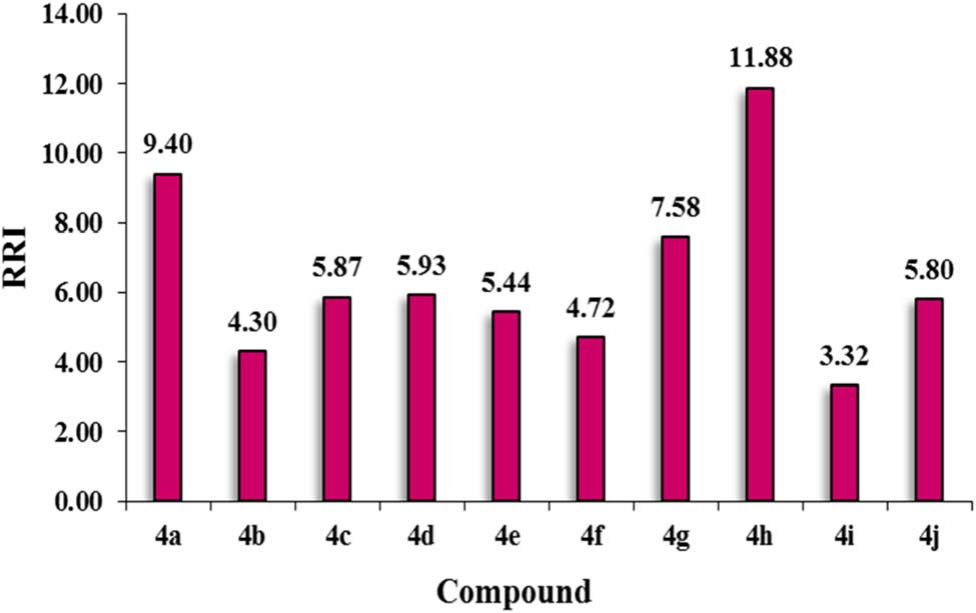
RRI values for the synthesized 1,8-dioxodecahydroacridine derivatives.

To evaluate the substrate scope of the M-NGO/UiO-66 catalyst in the synthesis of various derivatives, a series of substituted benzaldehydes and anilines were examined under standard reaction conditions (Table 3). Experimental results revealed that the reaction efficiency was strongly influenced by the electronic nature of the substituents present on both the benzaldehyde and the aniline. To provide a clearer semi-quantitative comparison of the reaction performance, the Relative Rate Index (RRI), defined as RRI = yield (%)/time (min), was calculated for all synthesized derivatives and is presented in Fig. 8. In general, electron-withdrawing groups (EWGs) on the benzaldehyde and electron-donating groups (EDGs) on the aniline increase the electrophilicity and nucleophilicity of the reactants, respectively, thereby facilitating the progress of the reaction. Accordingly, reactions involving benzaldehydes bearing EWGs and anilines bearing EDGs (compounds 4a, 4g, and 4h) proceeded in shorter times (8 to 12 min) with high yields (91 to 95%), consequently affording the highest RRI values of 9.40, 7.58, and 11.88, respectively. In contrast, compound 4i, derived from a benzaldehyde with an EDG and an aniline with an EWG, exhibited the longest reaction time (25 min) and the lowest yield (83%), resulting in the lowest RRI value (3.32). Furthermore, compounds in which both aromatic rings bore either EWGs or EDGs (**4b–4f** and 4j) showed moderate reaction times, satisfactory yields, and consequently intermediate RRI values in the range of 4.30 to 5.93. These observations indicate that the presence of an appropriate balance between the electrophilicity of the aldehyde and the nucleophilicity of the aniline plays a decisive role in the overall efficiency of the reaction.

**Table 3:** Synthesis of 1,8-dioxodecahydroacridine derivatives catalyzed by M-NGO/UiO-66^a^

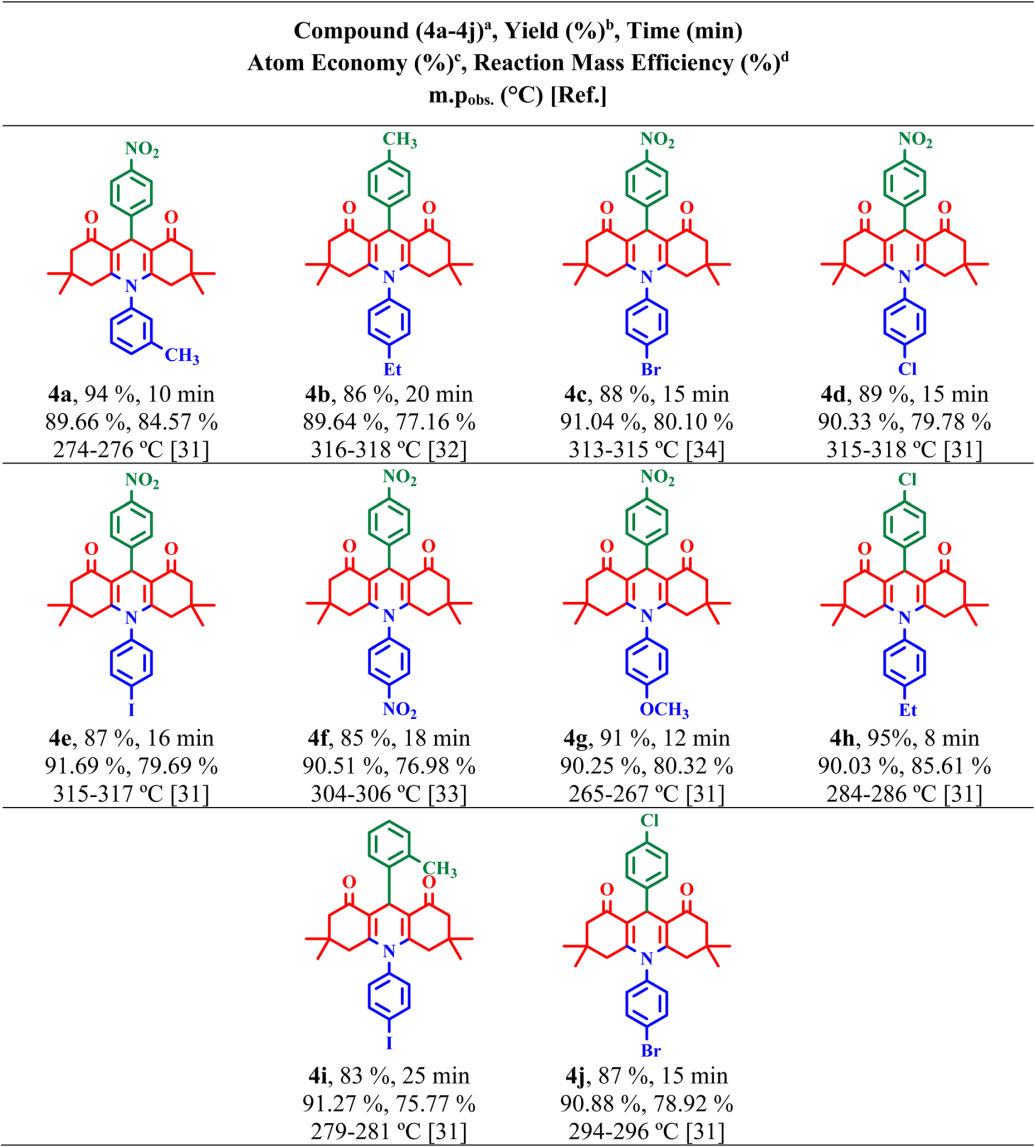

a Reaction conditions: dimedone (2 mmol), benzaldehyde derivatives (1 mmol), aniline derivatives (1 mmol), ethanol (3 mL), in the presence of 0.01 g of M-NGO/UiO-66 under reflux conditions.

b Isolated yield.

c Atom economy (AE): percentage ratio of the molecular weight of the desired product to the total molecular weight of all reactants.

d Reaction mass efficiency (RME): percentage ratio of the mass of the isolated product to the total mass of all reactants.

To further evaluate the environmental sustainability of the current protocol, green chemistry metrics, specifically AE and RME, were calculated for the synthesized derivatives (Table 2). Atom economy provides a measure of the reactant mass incorporated into the final product, while RME offers a more comprehensive assessment by accounting for the isolated yield.^83^ The high values obtained for both AE and RME indicate that the developed synthetic procedure is efficient and adheres to the principles of green chemistry.

### Proposed mechanism for the synthesis of 1,8-dioxodecahydroacridine derivatives

**Scheme 3:**
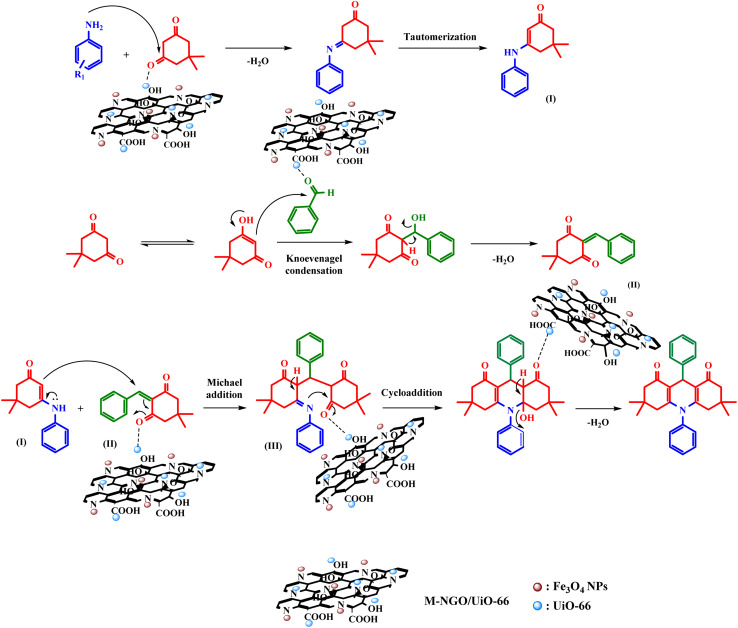
Plausible mechanism for the preparation of 1,8-dioxodecahydroacridine derivatives using M-NGO/UiO-66.

Based on the well-established mechanism for the synthesis of 1,8-dioxodecahydroacridines^31,84^ and the structural features of the M-NGO/UiO-66 nanocomposite, a plausible catalytic cycle is proposed (Scheme 3). The catalyst possesses multiple active sites, including Lewis acidic Zr^4+^ centers in the UiO-66 framework, as well as nitrogen-containing functional groups on the N-GO, as confirmed by FT-IR, XRD, EDS, and BET analyses. These sites enable effective dual activation of the reactants. The reaction begins with the activation of the carbonyl group of dimedone through coordination to the Lewis acidic Zr(iv) sites of catalyst. This increases the electrophilicity of the carbonyl carbon and facilitates nucleophilic attack by the aniline derivative, leading to the formation of enaminone intermediate I after dehydration and imine–enamine tautomerization. Concurrently, the catalyst activates the aldehyde, promoting its Knoevenagel condensation with a second molecule of dimedone to generate the α,β-unsaturated ketone intermediate II after removal of H_2_O. Intermediates I and II then undergo Michael addition, followed by intramolecular cyclization and dehydration to furnish the target 1,8-dioxodecahydroacridine derivative. The excellent catalytic performance of M-NGO/UiO-66 under mild conditions strongly supports the presented pathway.

### Investigation of the recovery and reusability of the M-NGO@UiO-66 nanocatalyst

**Fig. 9:**
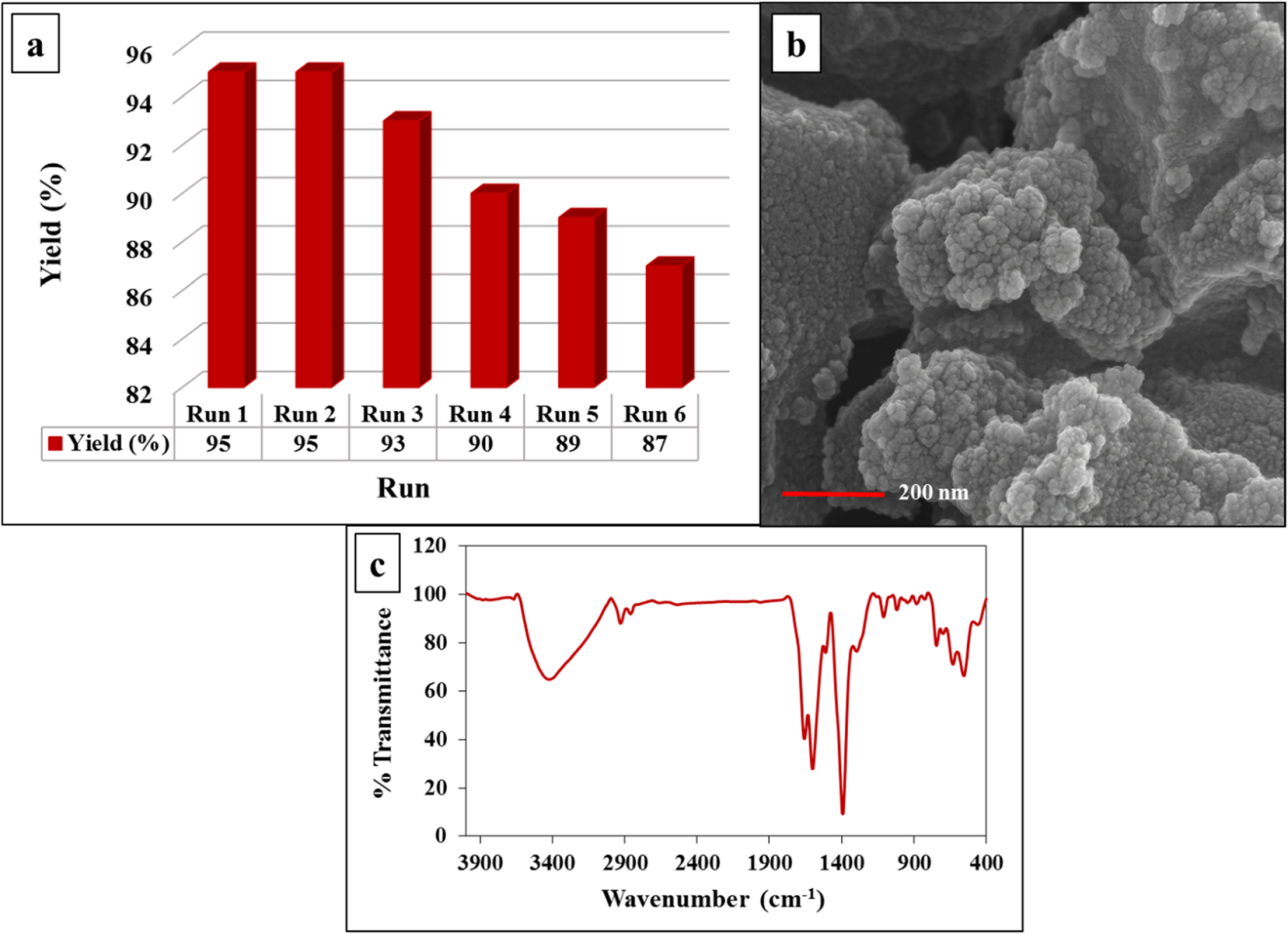
The reusability results of M-NGO/UiO-66 in the synthesis of 1,8-dioxodecahydroacridine (a), FE-SEM image (b) and FT-IR analysis (c) of M-NGO/UiO-66 after the 6th reuse.

One of the key advantages of heterogeneous catalysts is their ease of separation from the reaction medium and their potential for reuse in multiple synthesis cycles. To evaluate this critical performance characteristic, the stability and recyclability of the synthesized M-NGO/UiO-66 catalyst were assessed in the synthesis of 4h derivative. After each reaction, the catalyst was carefully separated from the reaction mixture, washed thoroughly with distilled water and ethanol, and dried at 80 °C for 6 hours. The recovered catalyst was then reused in identical reactions, (to six times). The results demonstrate that the catalytic activity remained consistently high, with no significant decline in performance across the cycles (Fig. 9). To further confirm the absence of changes in the catalyst's morphology and chemical structure after multiple reuse cycles, FE-SEM and FT-IR spectroscopy analyses were conducted on the catalyst after six consecutive cycles. Findings unequivocally indicated that the catalyst maintained excellent structural and chemical stability, retaining its effective performance even after six rounds of recycling.

### Catalytic performance comparison with previously reported catalysts

To compare the catalytic performance of the prepared M-NGO/UiO-66 nanocomposite with previously reported catalysts, the synthesis of compound 4d was selected as the model reaction. As illustrated in Table 4, the M-NGO/UiO-66 catalyst exhibited excellent efficiency, achieving 89% yield in only 15 min under mild and green conditions (ethanol as solvent, reflux). Although some previously reported methods afforded slightly higher yields, the present catalytic system offers significantly shorter reaction times compared to most of the other catalysts. Moreover, it operates with a very low catalyst loading (0.01 g), employs ethanol as an environmentally benign solvent, and benefits from facile magnetic separation, which are notable advantages over many existing protocols. These features make M-NGO/UiO-66 a highly efficient, green, and practical nanocatalyst for this transformation.

**Table 4:** Critical comparison of catalyst efficiency for compound 4d synthesis^a^

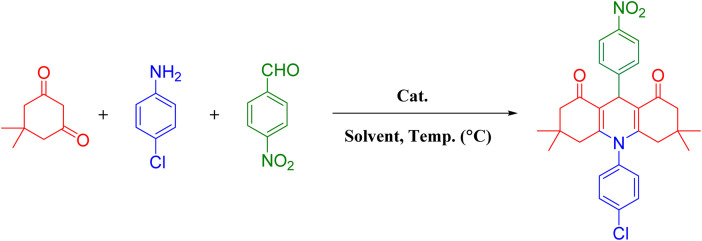					
Entry	Catalyst (amount)	Conditions	Time (min)	Yield^b^ (%)	Ref.
1	Vitamin B_1_ (10 mol%)	H_2_O, r.t	1440	83	85
2	[SEMSA]^c^ (0.1 g)	EtOH, 80 °C	20	92	86
3	Graphene oxide (0.01 g)	DMF, 100 °C	90	91	36
4	*p*-Dodecylbenzenesulphonic acid (10 mol%)	H_2_O, reflux	360	89	87
5	GO/KCC-1(0.16)/Ni(ii) (0.015 g)	H_2_O, 75 °C	150	75	31
6	[ImSi][PF_6_]@xanthan (50 mg)	EtOH, 80 °C	41	95	33
7	M-NGO@UiO-66 (0.01 g)	EtOH, reflux	15	89	This work

a Reaction conditions: dimedone (2 mmol), 4-nitrobenzaldehyde (1 mmol), 4-chloroaniline (1 mmol).

b Isolated yield.

c Cellulose [2-(sulfooxy)ethyl]mercaptosulfonic acid.

## Conclusion

The present study successfully demonstrates the synthesis and application of a novel nanocomposite based on N-doped graphene oxide (M-NGO/UiO-66) as a highly efficient and sustainable catalyst for the synthesis of 1,8-dioxodecahydroacridine derivatives. The integration of nitrogen-doped graphene oxide with Fe_3_O_4_ nanoparticles and UiO-66 MOF, was achieved through a controlled hydrothermal process, resulted in a robust nanocatalyst with exceptional structural stability, high surface area, and magnetic recoverability. Comprehensive characterization *via* FTIR, XRD, SEM, EDS, elemental mapping, BET, and VSM analyses confirmed the successful formation of the hybrid structure and its uniform elemental distribution. The catalyst exhibited outstanding performance, achieving 83–95% yield through 8–25 min under optimized green conditions (ethanol, reflux, 0.01 g of catalyst), surpassing previously reported catalysts in both efficiency and reaction time. The M-NGO/UiO-66 nanocatalyst's remarkable recyclability, maintaining consistent activity over six cycles without structural or chemical degradation, underscores its practical utility in sustainable catalysis. These findings highlight the potential of M-NGO/UiO-66 as a versatile, eco-friendly and magnetically separable catalyst, paving the way for its application in other organic transformations and advancing the field of green chemistry.

## Conflicts of interest

There are no conflicts to declare.

## Supplementary Material

RA-OLF-D6RA03005J-s001

## Data Availability

The data that supports the findings of this manuscript are available in the supporting information (SI). Supplementary information is available. See DOI: https://doi.org/10.1039/d6ra03005j.
