# Supplementary material for: M-NGO/UiO-66 nanocomposite: a green and efficient catalyst for the synthesis of 1,8-dioxodecahydroacridine derivatives
Source: RSC Adv. 2026 Jul 17;16(37):40896–909. doi: 10.1039/d6ra03005j (PMC13377563; doi:10.1039/d6ra03005j)
Supplement: Supplementary file 1 [file RA-016-D6RA03005J-s001.pdf]

Supplementary Information (SI) for RSC Advances.
This journal is © The Royal Society of Chemistry 2026

M-NGO/UiO-66 nanocomposite: a green and efficient catalyst for the synthesis of 1,8-dioxodecahydroacridine derivatives

Atefeh Zeinali, Leila Moradi*

Department of organic chemistry, faculty of chemistry, university of Kashan, P.O. Box 8731753153, Kashan, I.R.Iran

**Corresponding author*. E-mail address: l_moradi@kashanu.ac.ir

Spectral data

10-(3-methylphenyl)-3,3,6,6-tetramethyl-9-(4-nitrophenyl)-3,4,6,7,9,10-hexahydroacridine-1,8(2H,5H)-dione (4a)

M.P. =274-276 ºC [29]; FTIR (KBr) ($\bar{\upsilon}$/cm^{-1}): 3427, 2956, 1634, 1512, 1457, 1357, 1224, 1143, 1006, 832, 744, 689; ^{1}H NMR (400 MHz, DMSO-d_6, δ, ppm): 8.15 (d, J = 8.1 Hz, 2H, ArH), 7.57 (d, J = 8.3 Hz, 2H, ArH), 7.49 (s, 1H, ArH), 7.38 (m, 1H, ArH) 7.28 (m, 2H, ArH), 5.12 (s, 1H, CH), 2.7 (s, 3H, CH_3), 2.19 (d, J = 16.8 Hz, 4H, 2 CH_2), 1.99 (d, J = 16.2 Hz, 2H, CH_2), 1.79 (d, J = 17.6 Hz, 2H, CH_2), 0.87 (s, 6H, 2CH_3), 0.69 (s, 6H, 2CH_3).

10-(4-ethylphenyl)-3,3,6,6-tetramethyl-9-(p-tolyl)-3,4,6,7,9,10-hexahydroacridine-1,8(2H,5H)-dione (4b)

M.P. =316-318 ºC [30]; FTIR (KBr) ($\bar{\upsilon}$ /cm^{-1}): 3245, 3178, 3101, 2926, 1572, 1527, 1413, 1274, 1147, 815, 714; ^{1}H NMR (400 MHz, $CDCl_3$, δ, ppm): 7.28 (s, 3H, ArH), 7.19 (d, J = 8.0 Hz, 2H, ArH), 7.11 (d, J = 8.0 Hz, 3H, ArH), 5.63 (s, 1H, CH), 2.65 (q, J = 7.6 Hz, 4H), 2.41 (s, 3H), 2.27 (s, 3H), 1.25 (dd, J = 14.2, 12.0 Hz, 4H), 1.13 (s, 6H).

10-(4-bromophenyl)-3,3,6,6-tetramethyl-9-(4-nitrophenyl)-3,4,6,7,9,10-hexahydroacridine-1,8(2H,5H)-dione (Compound 4c)

M.P. =313-315 ºC [32]; FTIR (KBr) ($\bar{\upsilon}$/cm^{-1}): 3431, 2924, 2858, 1637, 1528, 1513, 1466, 1222, 1146, 1009, 853, 740, 696; ^{1}H NMR (400 MHz, $CDCl_3$, δ, ppm): 8.15 (d, J = 8.0 Hz, 2H, ArH), 7.75 (d, J = 8.0 Hz, 2H, ArH), 7.60 (d, J = 8.0 Hz, 2H, ArH), 7.15 (d, J = 8.0 Hz, 2H, ArH), 5.36 (s, 1H, CH), 2.26-2.13 (m, 7H), 2.09 (d, J = 16.0 Hz, 2H, ArH), 1.86 (d, J = 16.0 Hz, 2H, ArH), 0.88 (s, 6H), 0.83 (s, 6H).

10-(4-chlorophenyl)-3,3,6,6-tetramethyl-9-(4-nitrophenyl)-3,4,6,7,9,10-hexahydroacridine-1,8(2H,5H)-dione (Compound 4d)

M.P. =315-318 ºC [29]; FTIR (KBr) (ʋ/cm^{-1}): 3440, 2925, 2859, 1639, 1584, 1514, 1359, 1223, 1148, 1099, 1012, 857; ^{1}H NMR (400 MHz, $CDCl_3$, δ, ppm): 8.15 (d, J = 8.4 Hz, 1H, ArH), 7.60 (d, J = 8.4 Hz, 3H, ArH), 7.28 (s, 3H, ArH), 7.22 (d, J = 6.6 Hz, 1H, ArH), 5.36 (s, 1H, CH), 2.26 – 2.08 (m, 5H), 1.86 (d, J = 17.4 Hz, 2H), 1.62 (s, 1H), 1.28 (s, 6H), 0.99 (s, 6H).

10-(4-iodophenyl)-3,3,6,6-tetramethyl-9-(4-nitrophenyl)-3,4,6,7,9,10-hexahydroacridine-1,8(2H,5H)-dione (Compound 4e)

M.P. =315-317 ºC [29]; FTIR (KBr) (ʋ/cm^{-1}): 3432, 3064, 2927, 2537, 1637, 1513, 1460, 1271, 1155, 1055, 951, 827, 759; ^{1}H NMR (400 MHz, $CDCl_3$, δ, ppm): 8.15 (d, J = 8.0 Hz, 2H, ArH), 7.95 (s, 2H, ArH), 7.60 (d, J = 8.0 Hz, 2H, ArH), 5.36 (s, 1H, ArH), 7.02 (s, 2H, CH), 2.19-1.74 (m, 8H), 1.00 (s, 6H), 0.83 (s, 6H).

3,3,6,6-tetramethyl-9,10-bis(4-nitrophenyl)-3,4,6,7,9,10-hexahydroacridine-1,8(2H,5H)-dione (Compound 4f)

M.P. =304-306 ºC [31]; FTIR (KBr) (ʋ/cm^{-1}): 3474, 3066, 2870, 1639, 1590, 1522, 1351, 1294, 1223, 1149, 1011, 864, 700; ^{1}H NMR (400 MHz, $CDCl_3$, δ, ppm): 8.51 (d, J = 8.0 Hz, 2H, ArH), 8.17 (d, J = 8.0 Hz, 2H, ArH), 7.61 (d, J = 8.0 Hz, 2H, ArH), 7.51 (d, J = 8.0 Hz, 2H, ArH), 5.37 (s, 1H, CH), 2.27-2.07 (m, 6H), 8.51 (d, J = 20.0 Hz, 2H), 1.61 (s, 2H), 1.00 (s, 6H, CH_3), 0.84 (s, 6H, CH_3).

3,3,6,6-Tetramethyl-9-(4-nitrophenyl) 10-(4-methoxyphenyl) 1,8-dioxodecahydroacridine (Compound 4g)

M.P. =265-267 ºC [29]; FTIR (KBr) (ʋ/cm^{-1}): 3419, 2957, 1640, 1579, 1513, 1467, 1363, 1348, 1295, 1248, 1222, 1174, 1144, 1107, 1025, 1003,887, 862, 832, 750, 701, 619, 568, 538, 446; ^{1}H NMR (400 MHz, DMSO-d_6, δ, ppm): 8.14 (d, J = 8.3 Hz, 2H, ArH), 7.56 (d, J = 8.3 Hz, 2H, ArH), 7.38 (d, J = 8.3 Hz, 2H, ArH), 7.12 (d, J = 8.1 Hz, 2H, ArH), 5.11 (s, 1H, CH), 3.85 (s, 3H, O–CH_3), 2.20 (dd, J = 16.7, 6.7 Hz, 4H, 2CH_2), 1.99 (d, J =16.0 Hz, 2H, CH_2), 1.83 (d, J = 16.2 Hz, 2H, CH_2), 0.87 (s, 6H, 2CH_3), 0.69 (s, 6H, 2CH_3).

3,3,6,6-Tetramethyl 9-(4-chlorophenyl)-10-(4-ethylphenyl) 1,8-dioxodecahydroacridine (Compound 4h)

M.P. =284-286 ºC [29]; FTIR (KBr) (ῡ/cm $^{-1}$): 3472, 2958, 2871, 1638, 1573, 1513, 1465, 1363, 1344, 1306, 1265, 1224, 1177, 1147, 1112, 1005, 886, 859, 829, 747, 700, 615, 570, 535, 425; ^{1}H NMR (400 MHz, DMSO-d6, δ, ppm): 8.14 (d, *J* = 8.3 Hz, 2H, ArH), 7.56 (d, *J* = 8.3 Hz, 2H, ArH), 7.44 (d, *J* = 8.0 Hz, 2H, ArH), 7.36 (d, *J* = 7.7 Hz, 2H, ArH), 5.12 (s, 1H, CH), 2.73 (q, *J* = 7.6 Hz, 2H, CH_2), 2.20 (dd, *J* = 16.9, 4.9 Hz, 4H, $2CH_2$), 1.98 (d, *J* =16.1 Hz, 2H, CH_2), 1.77 (d, *J* = 17.6 Hz, 2H, CH_2), 1.26 (t, *J* = 7.6 Hz, 3H, CH_3), 0.86 (s, 6H, $2CH_3$), 0.69 (s, 6H, $2CH_3$).

3,3,6,6-Tetramethyl 9-(2-methylphenyl)-10-(4-iodophenyl) 1,8-dioxodecahydroacridine (Compound 4i)

M.P. =279-281 ºC [29]; FTIR (KBr) (ῡ/cm^{-1}): 3430, 3049, 2955, 2877, 1638, 1577, 1483, 1364, 1299, 1261, 1221, 1176, 1145, 1057, 1007, 885, 847, 810, 764, 703, 569, 520, 462; ^{1}H NMR (400 MHz, DMSO-d_6, δ, ppm): 7.96 (d, J = 8.0 Hz, 2H, ArH), 7.23–7.02 (m, 5H, ArH), 6.90 (d, J = 7.3 Hz, 1H, ArH), 4.98 (s, 1H, CH), 2.25 (s, 3H, CH_3), 2.19 (d, *J* = 6.8 Hz, 2H, CH_2), 2.17 (d, *J* = 6.7 Hz, 2H, CH_2), 1.99 (d, *J* = 16.1 Hz, 2H, CH_2), 1.76 (d, *J* = 17.5 Hz, 2H, CH_2), 0.87 (s, 6H, $2CH_3$), 0.71 (s, 6H,$2CH_3$); ^{13}C NMR (100 MHz, DMSO-d_6, δ, ppm): 195, 152.5, 150.3, 145.2, 135.5, 129, 128.4, 127.1, 120, 110, 48.8, 40.8, 31.5, 30.3, 27.8, 25.2, 21.2.

3,3,6,6-Tetramethyl 9-(4-chlorophenyl)-10-(4-bromophenyl) 1,8-dioxodecahydroacridine (Compound 4j)

M.P. =294-296 ºC [29]; FTIR (KBr) (ῡ/cm^{-1}): 3554, 3459, 2955, 2869, 1640, 1579, 1483, 1363, 1297, 1262, 1222, 1176, 1147, 1120, 1095, 1059, 1007, 943, 888, 843, 777, 733, 711, 661, 624, 597, 569, 518, 458; ^{1}H NMR (400 MHz, DMSO-d_6, δ, ppm): 7.96 (d, *J* = 7.7 Hz, 2H, ArH), 7.28 (m, 6H, ArH), 4.99 (s, 1H, CH), 2.17 (d, *J* = 17.0 Hz, 4H, $2CH_2$), 1.99 (d, *J* = 16.1 Hz, 2H, CH_2), 1.76 (d, *J* = 17.7 Hz, 2H, CH_2), 0.87 (s, 6H, $2CH_3$), 0.70 (s, 6H, $2CH_3$).

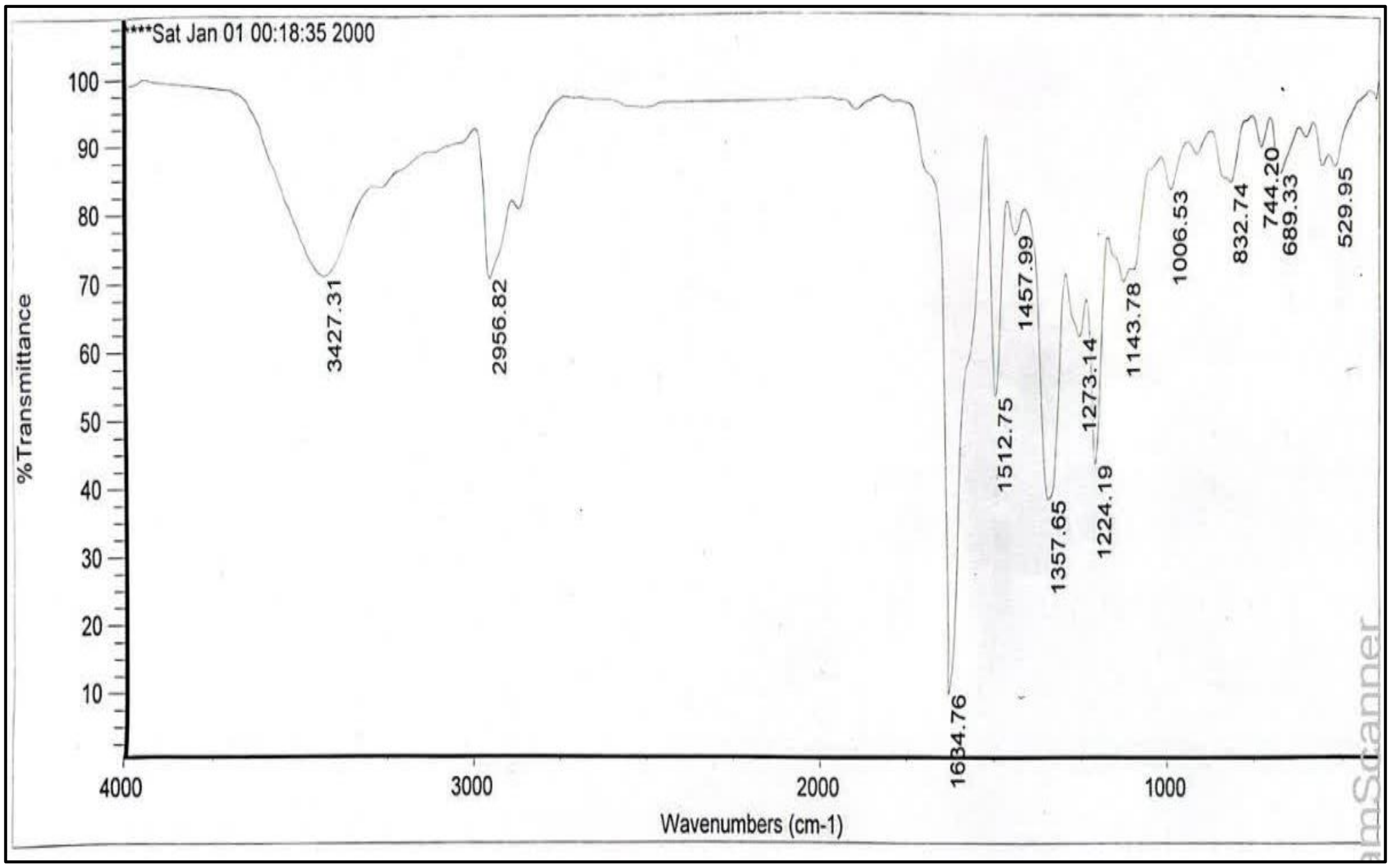

FT-IR of **4a**

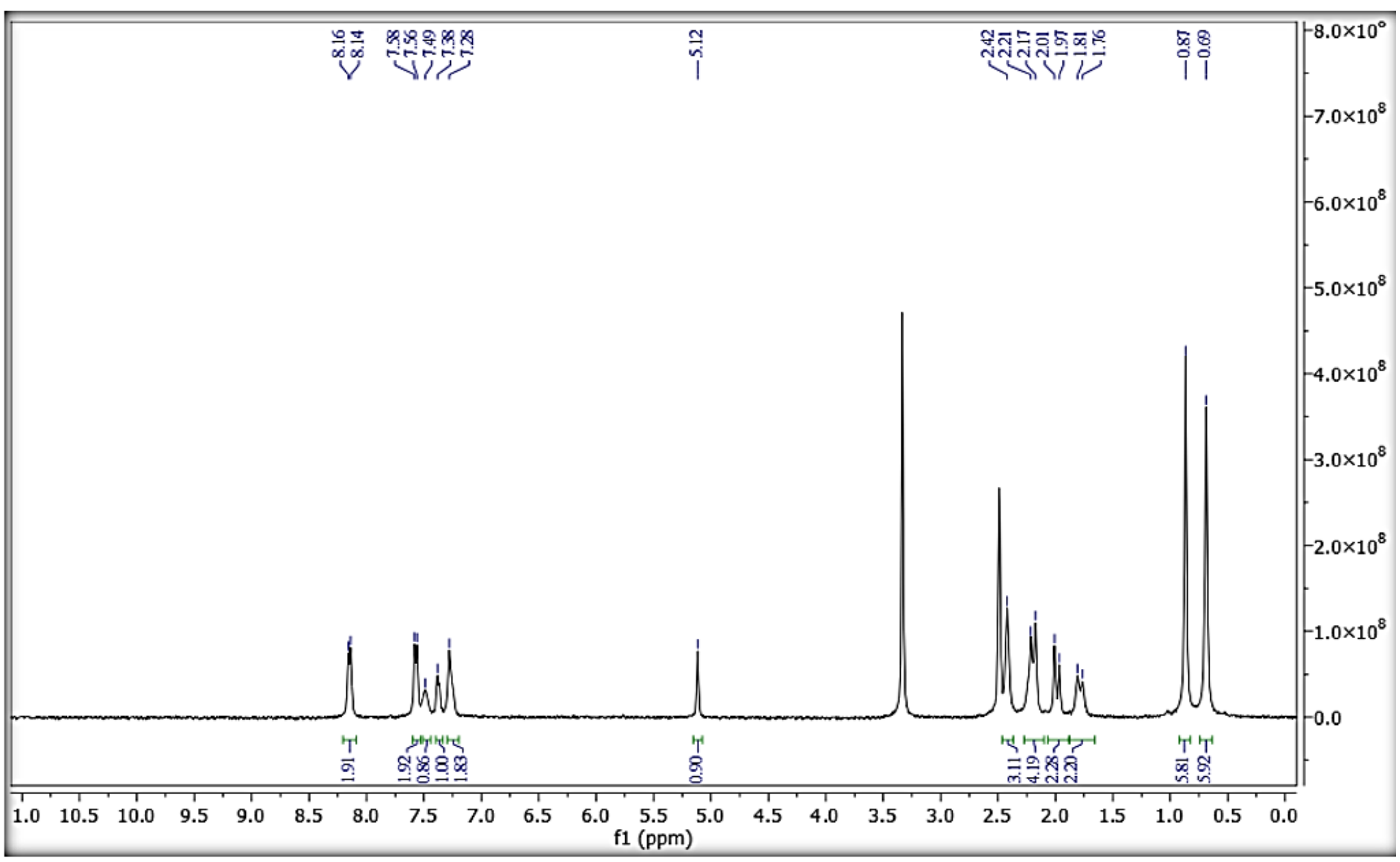

^{1}H NMR of **4a**

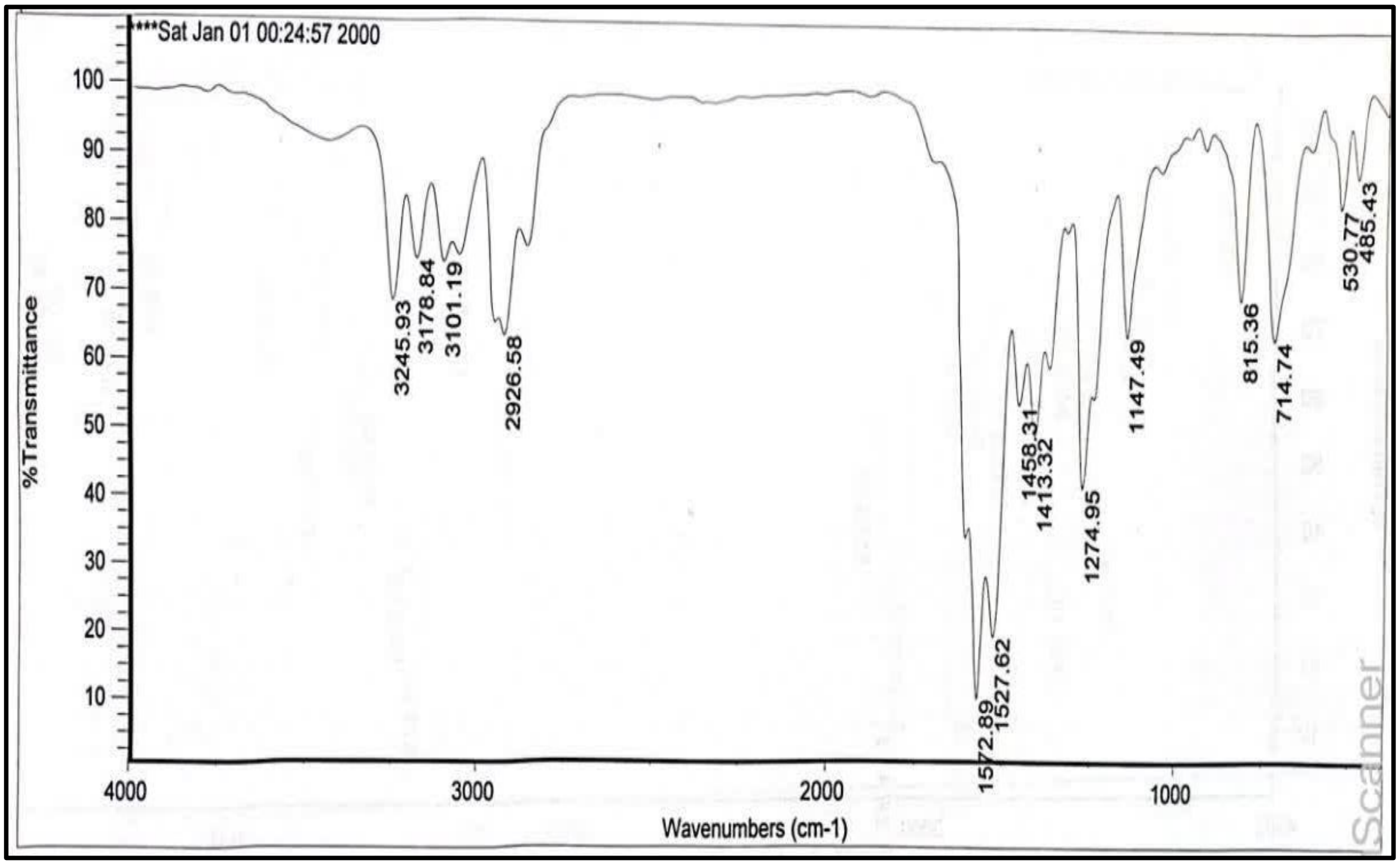

FT-IR of **4b**

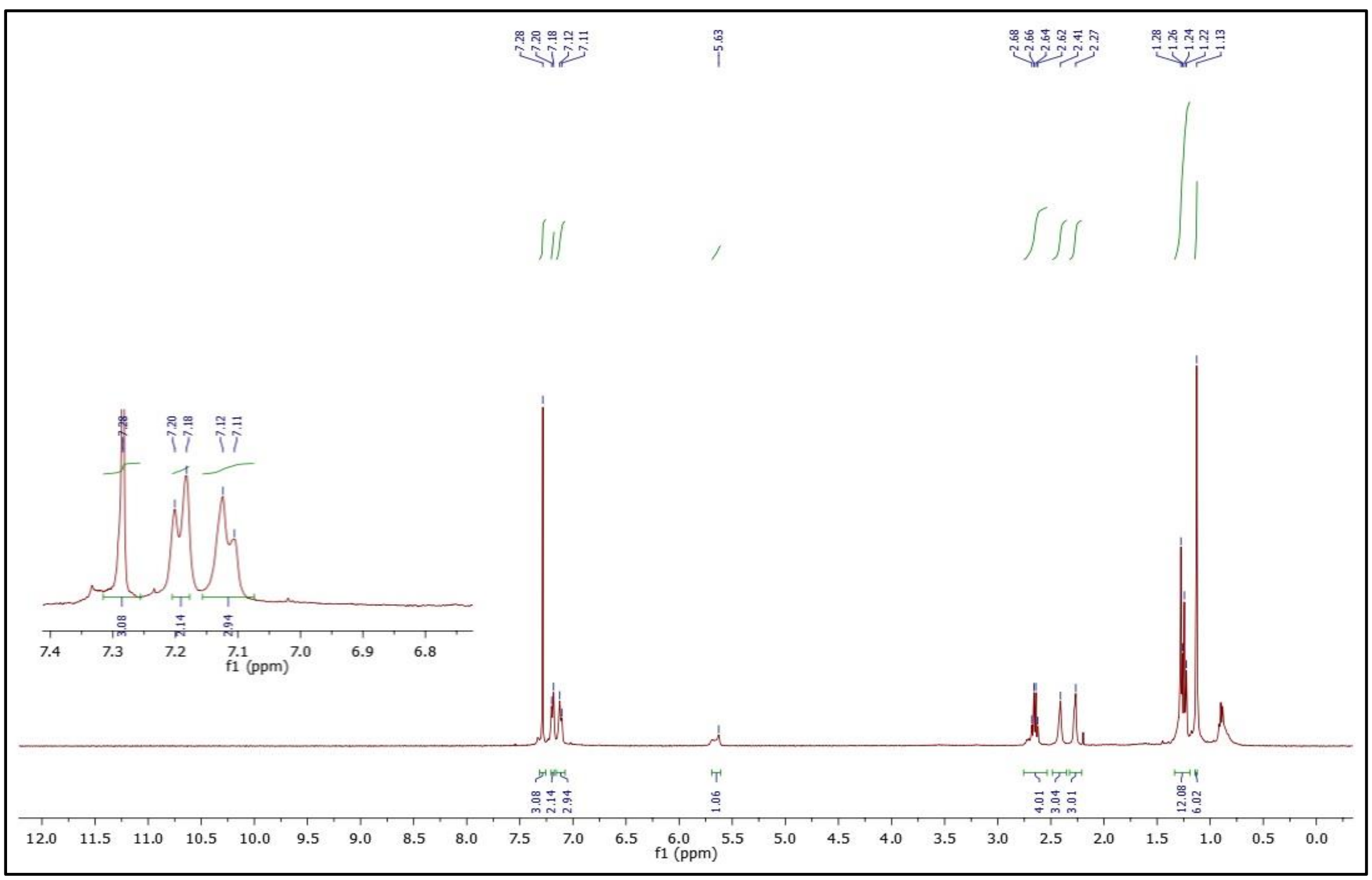

^{1}H NMR of **4b**

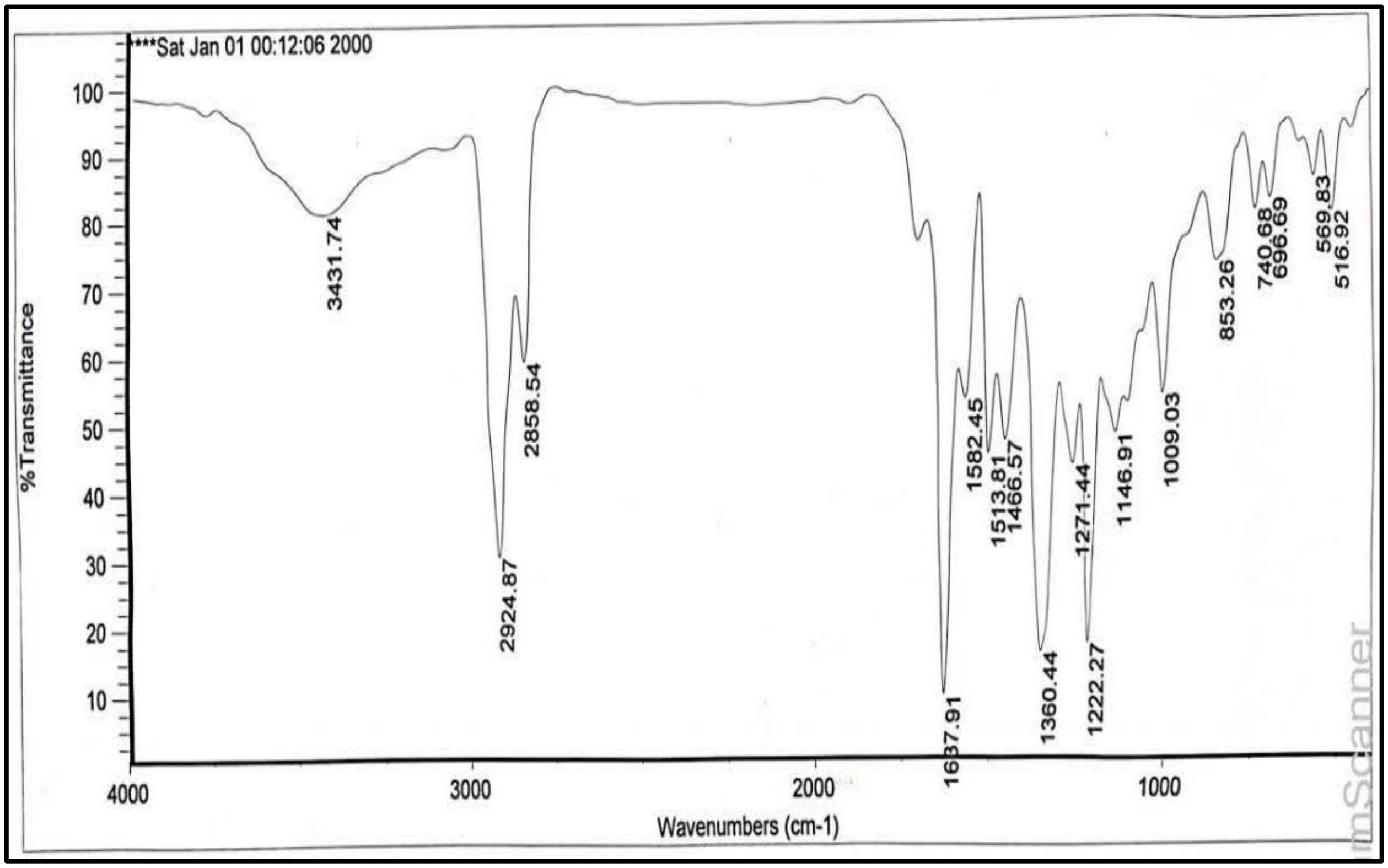

FT-IR of **4c**

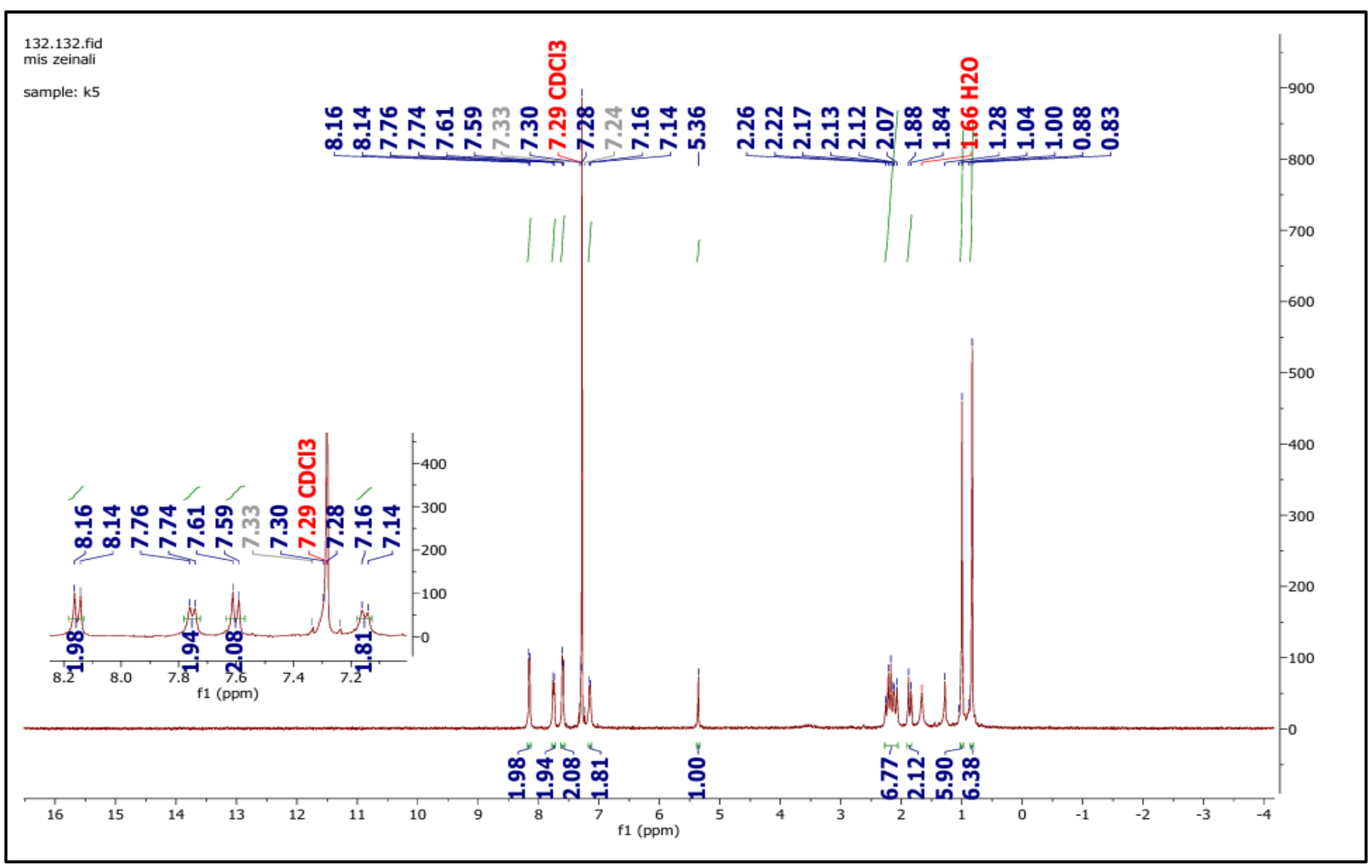

^{1}H NMR of **4c**

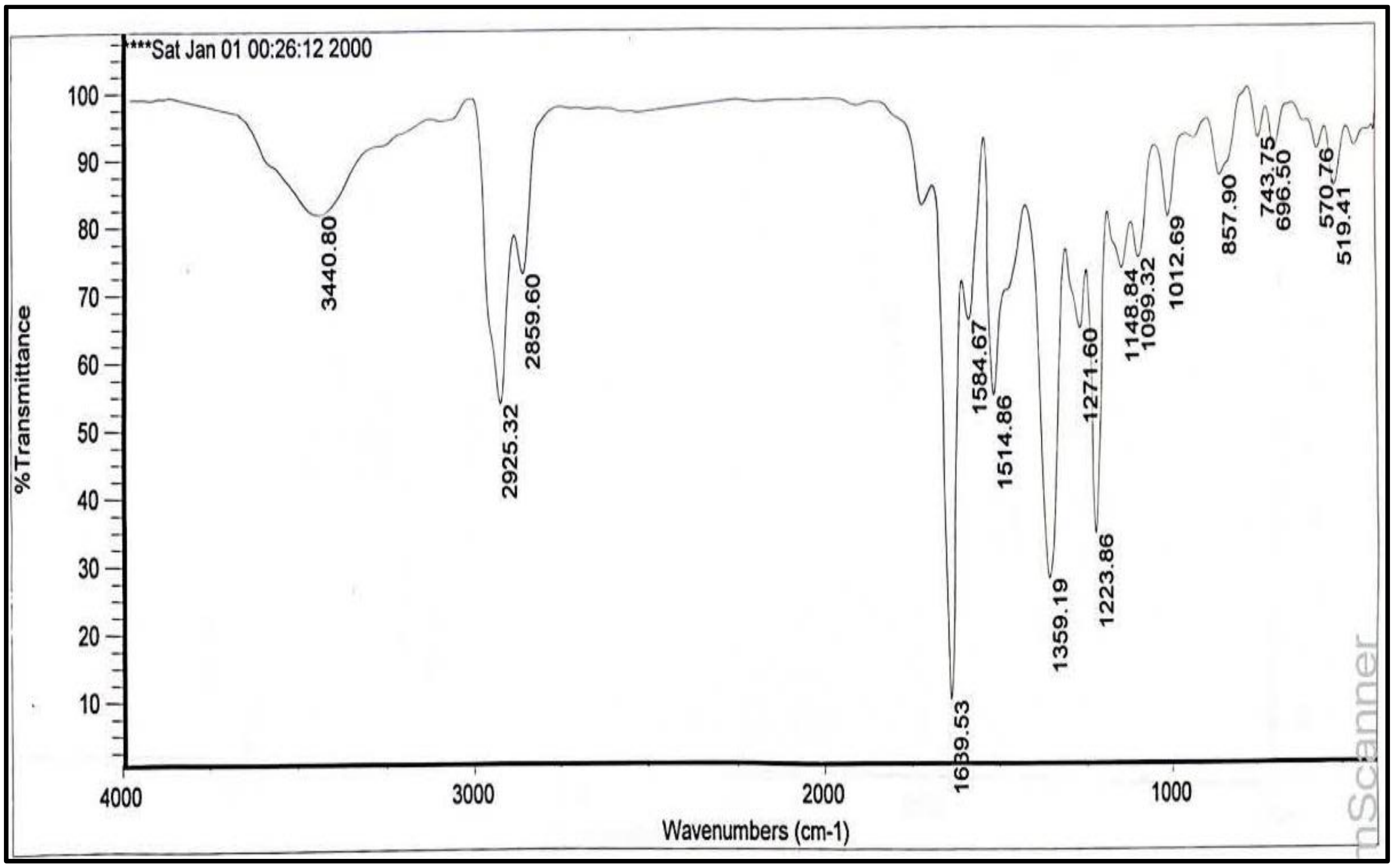

FT-IR of **4d**

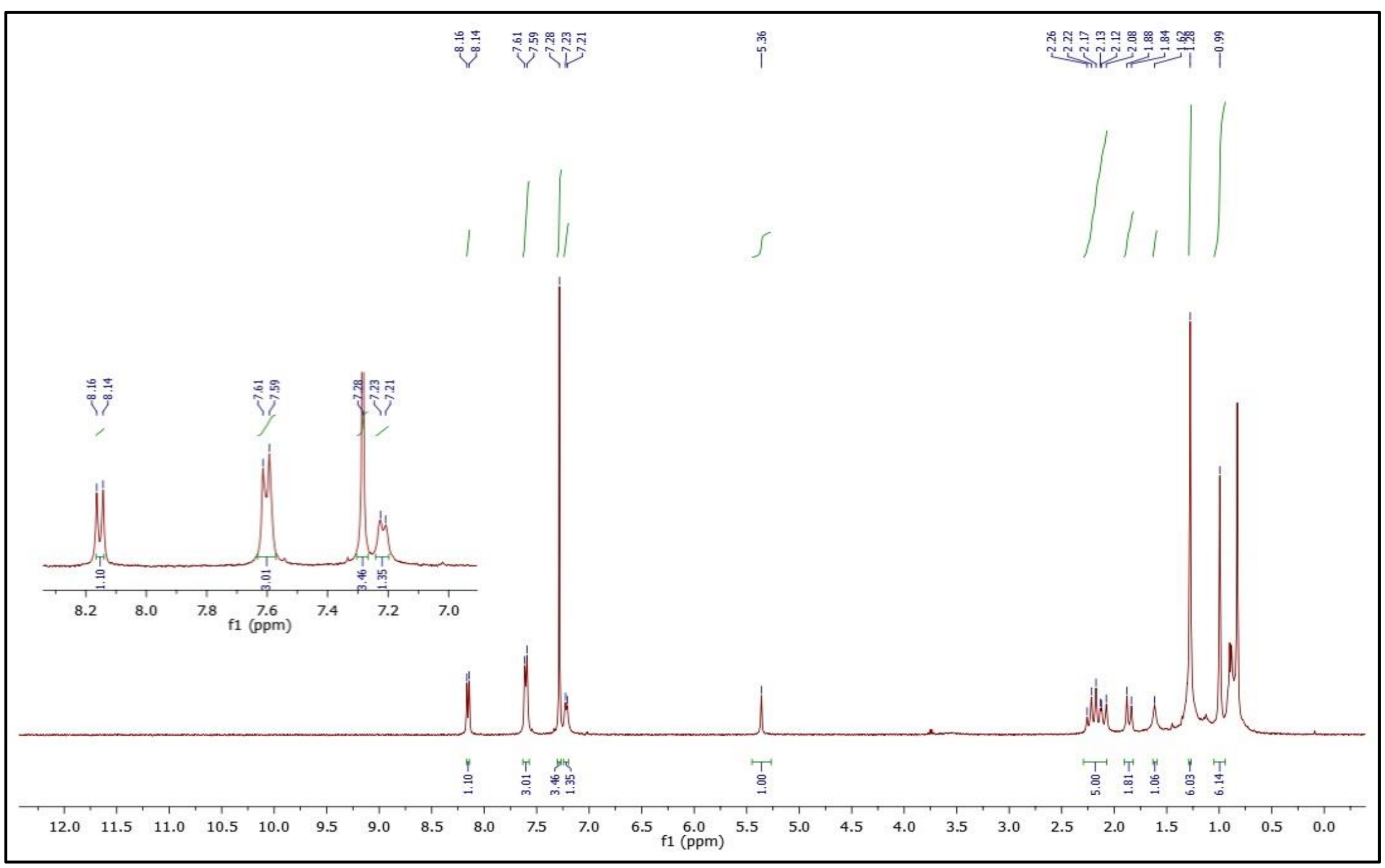

^{1}H NMR of **4d**

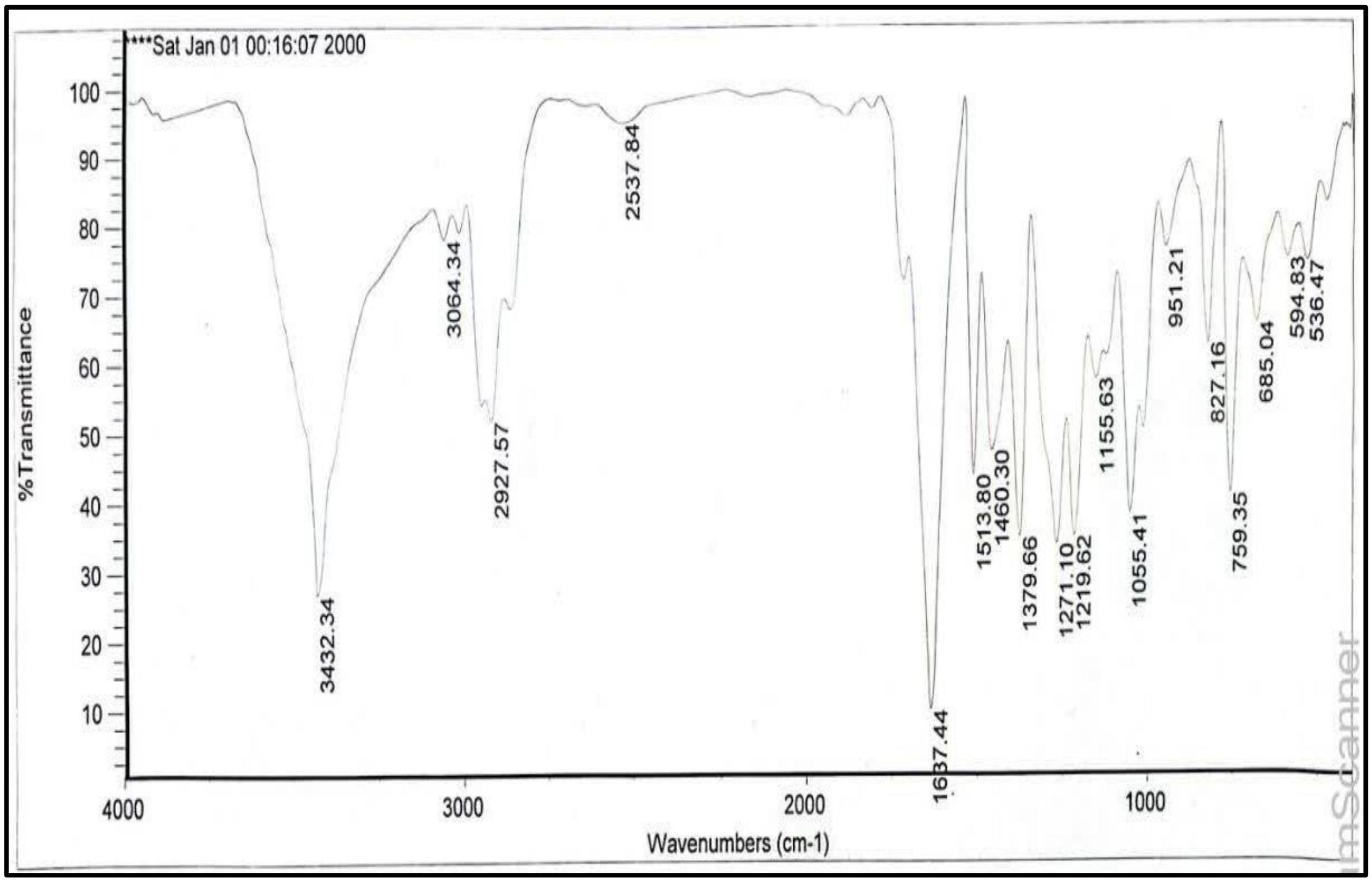

FT-IR of **4e**

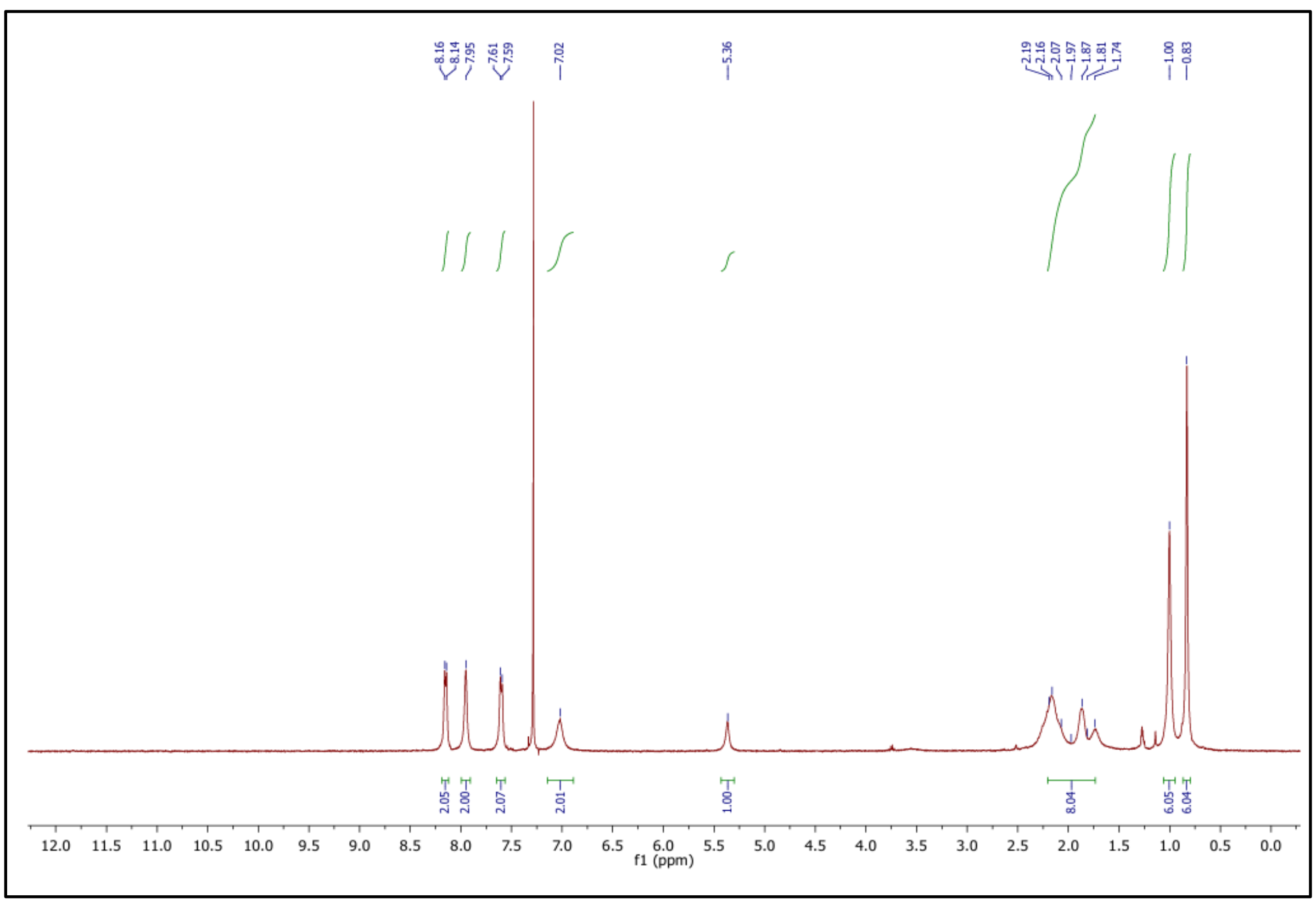

^{1}H NMR of **4e**

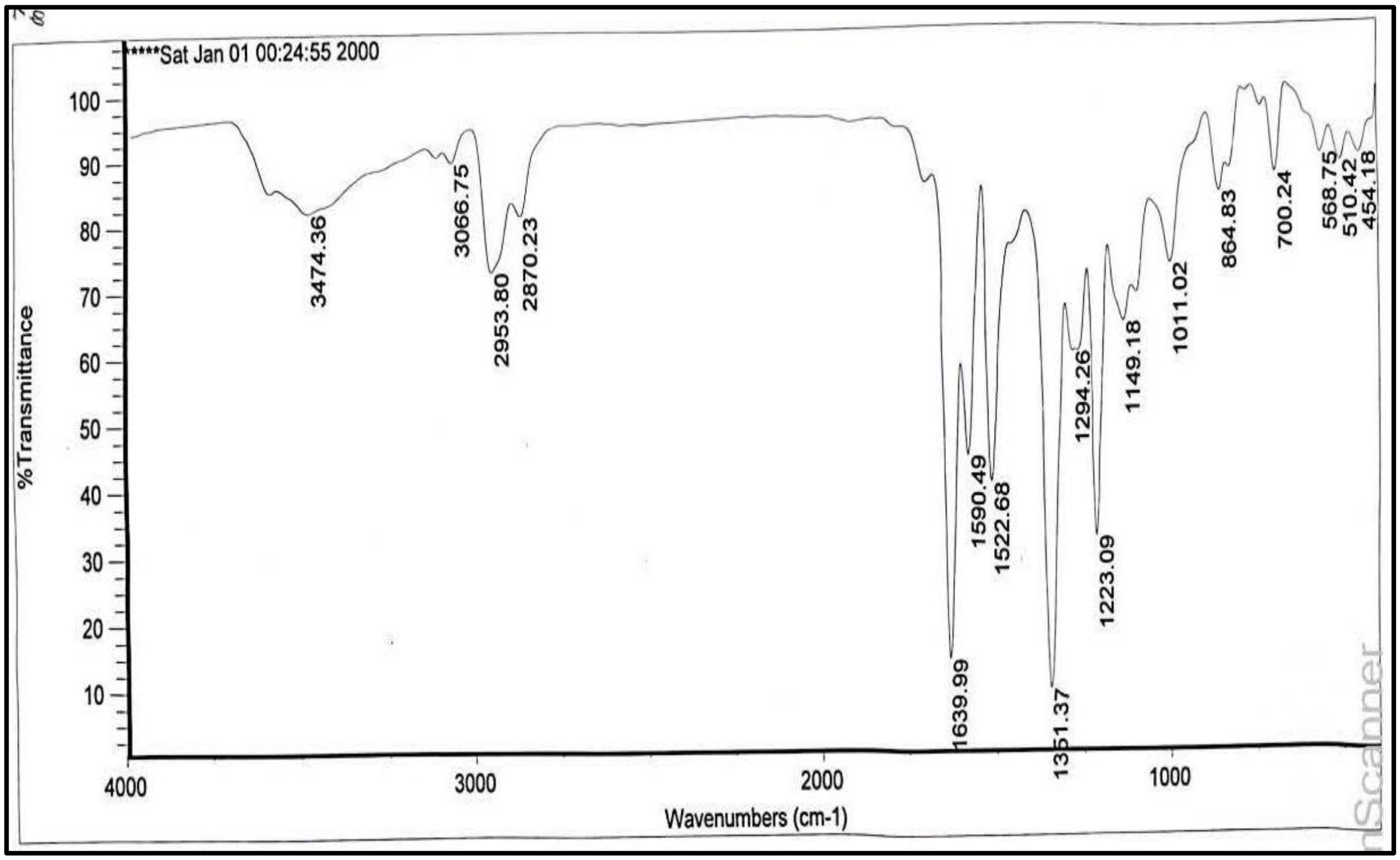

FT-IR of **4f**

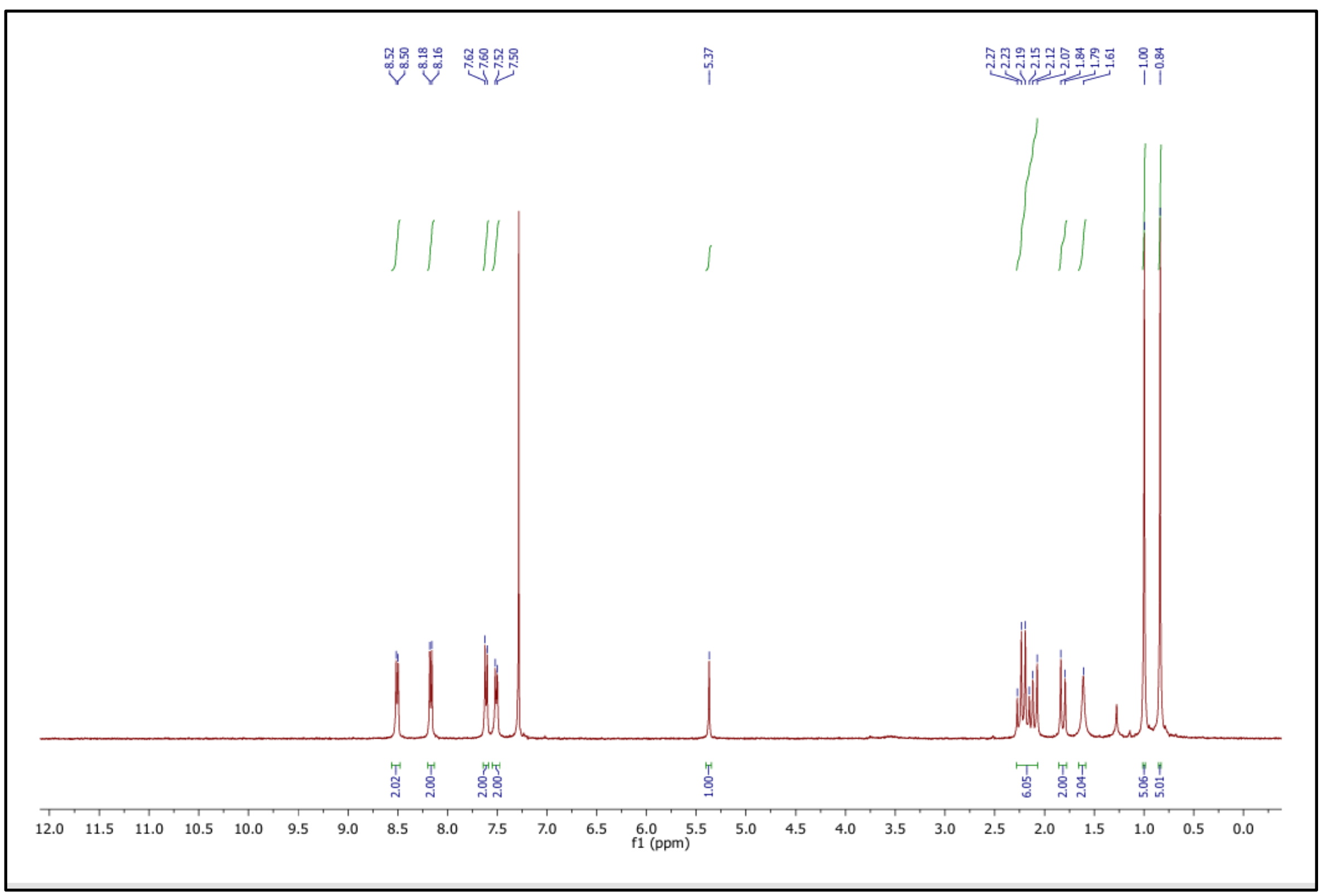

^{1}H NMR of **4f**

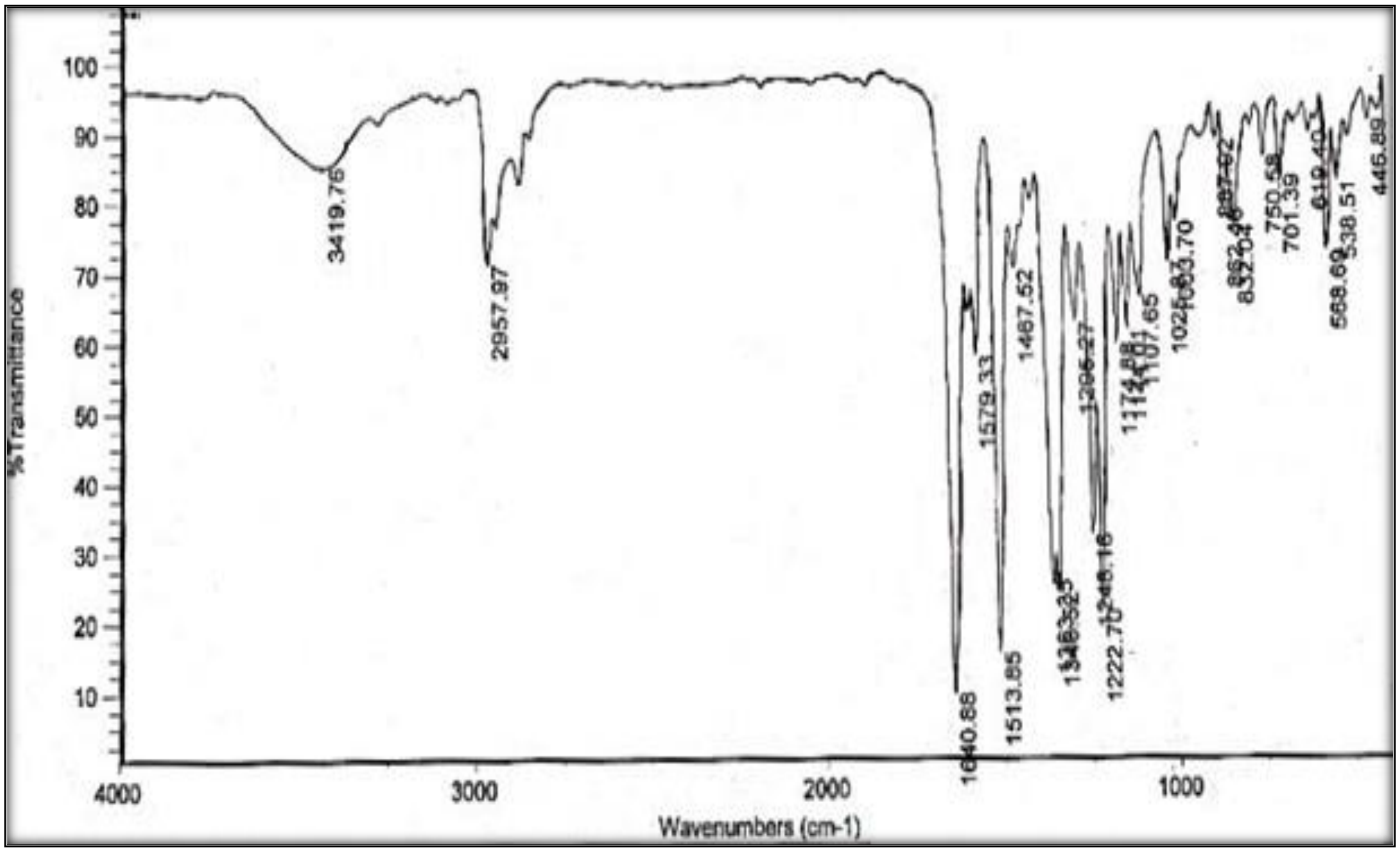

FT-IR of **4g**

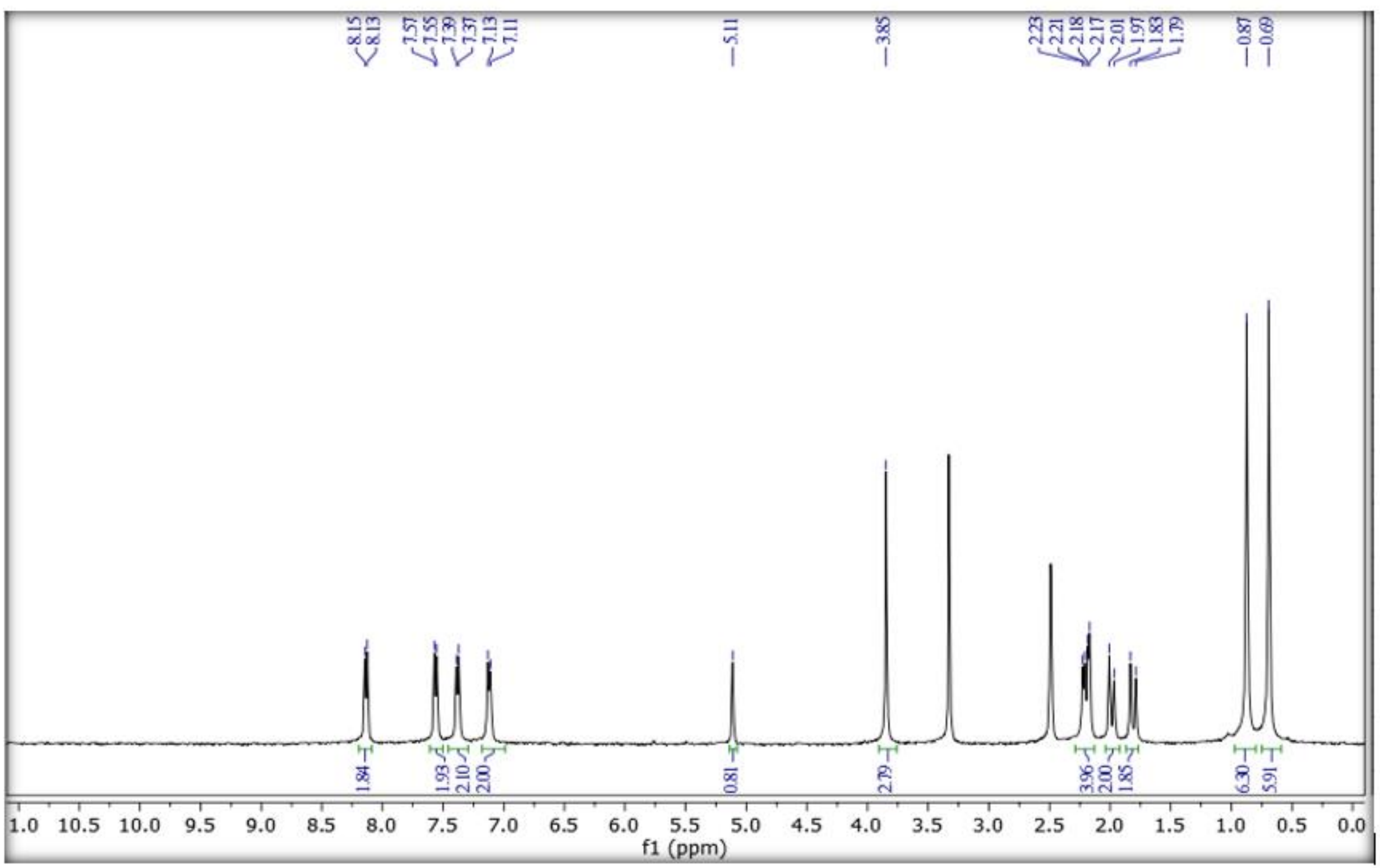

^{1}H NMR of **4g**

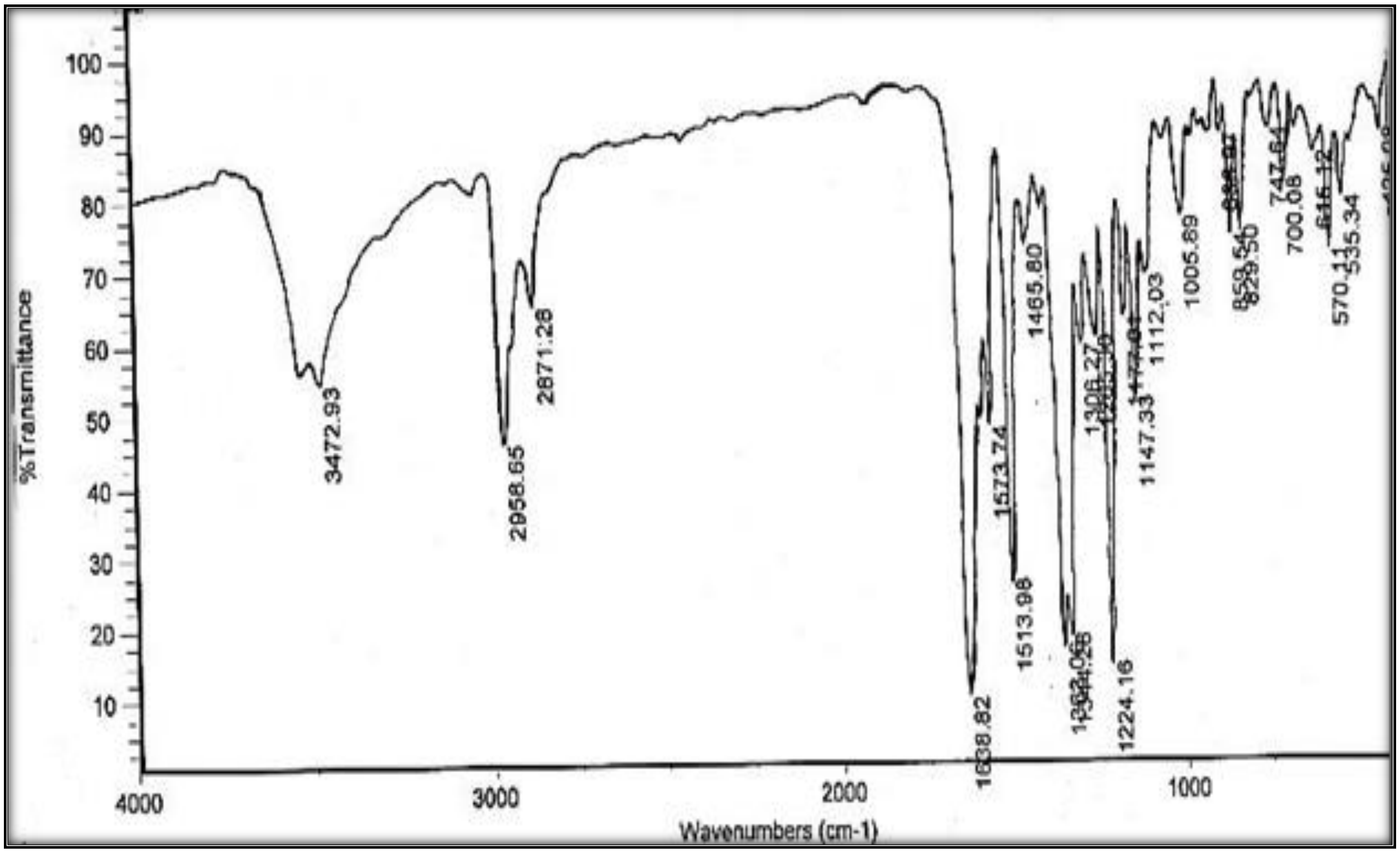

FT-IR of **4h**

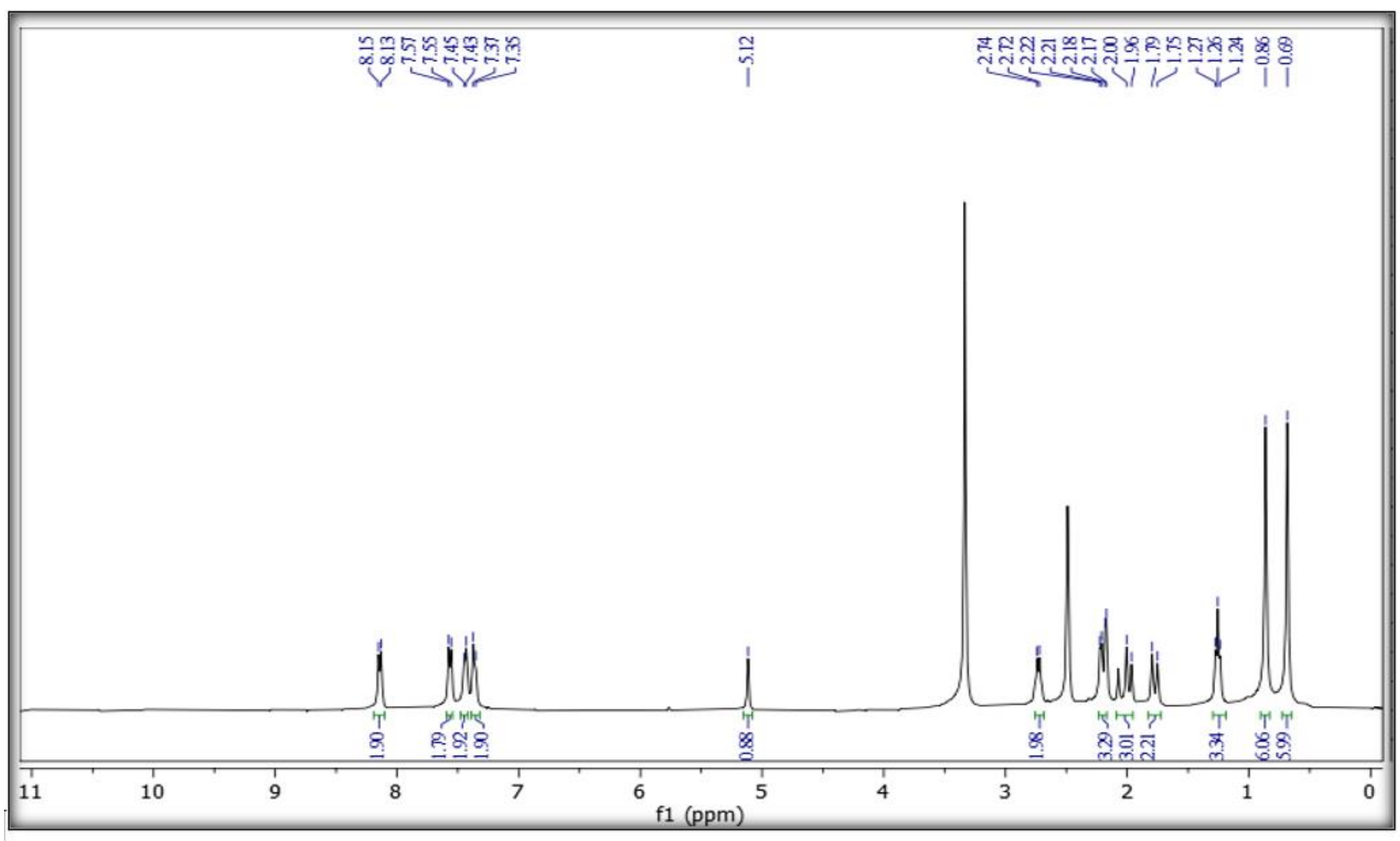

^{1}H NMR of **4h**

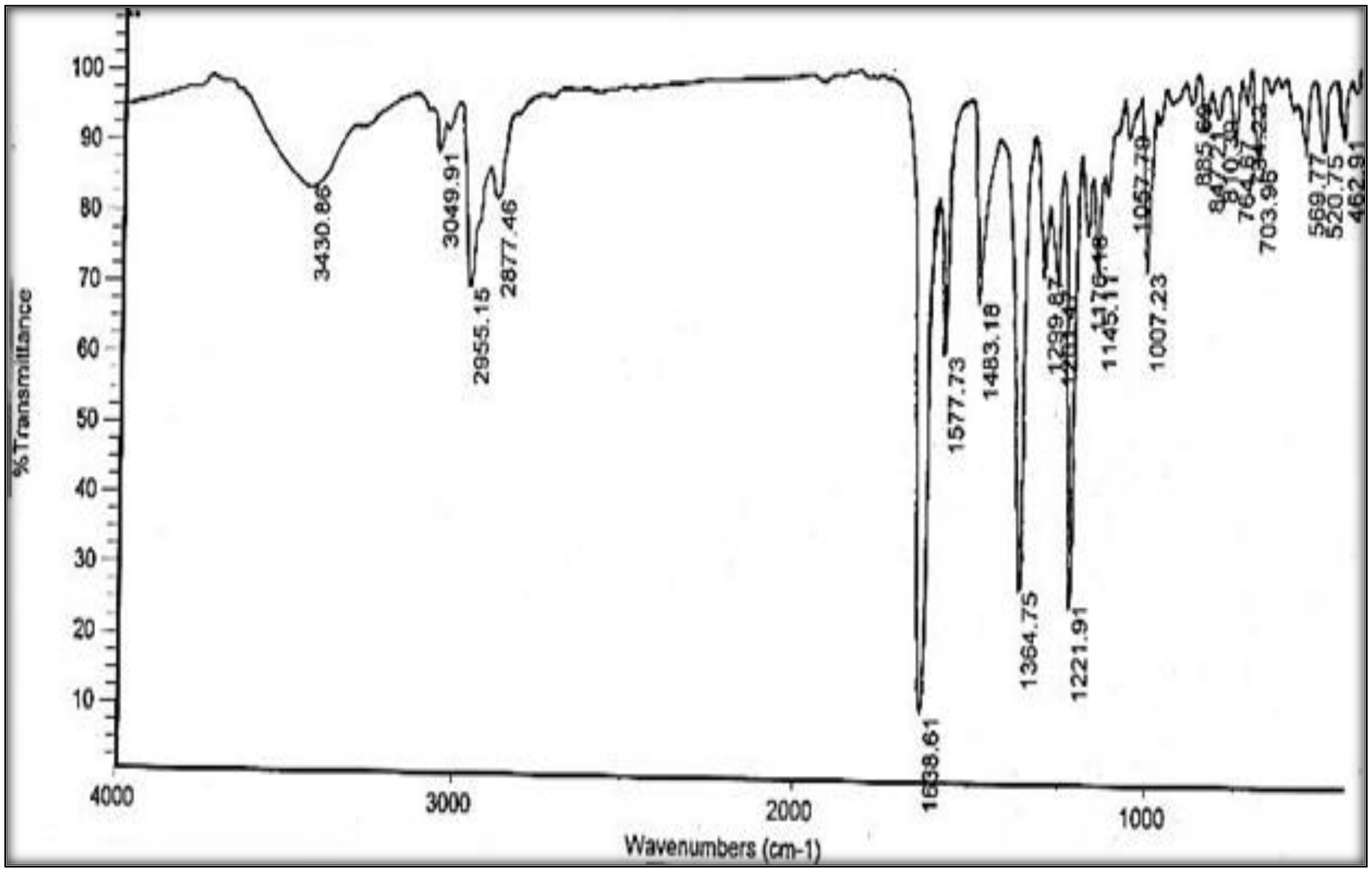

FT-IR of **4i**

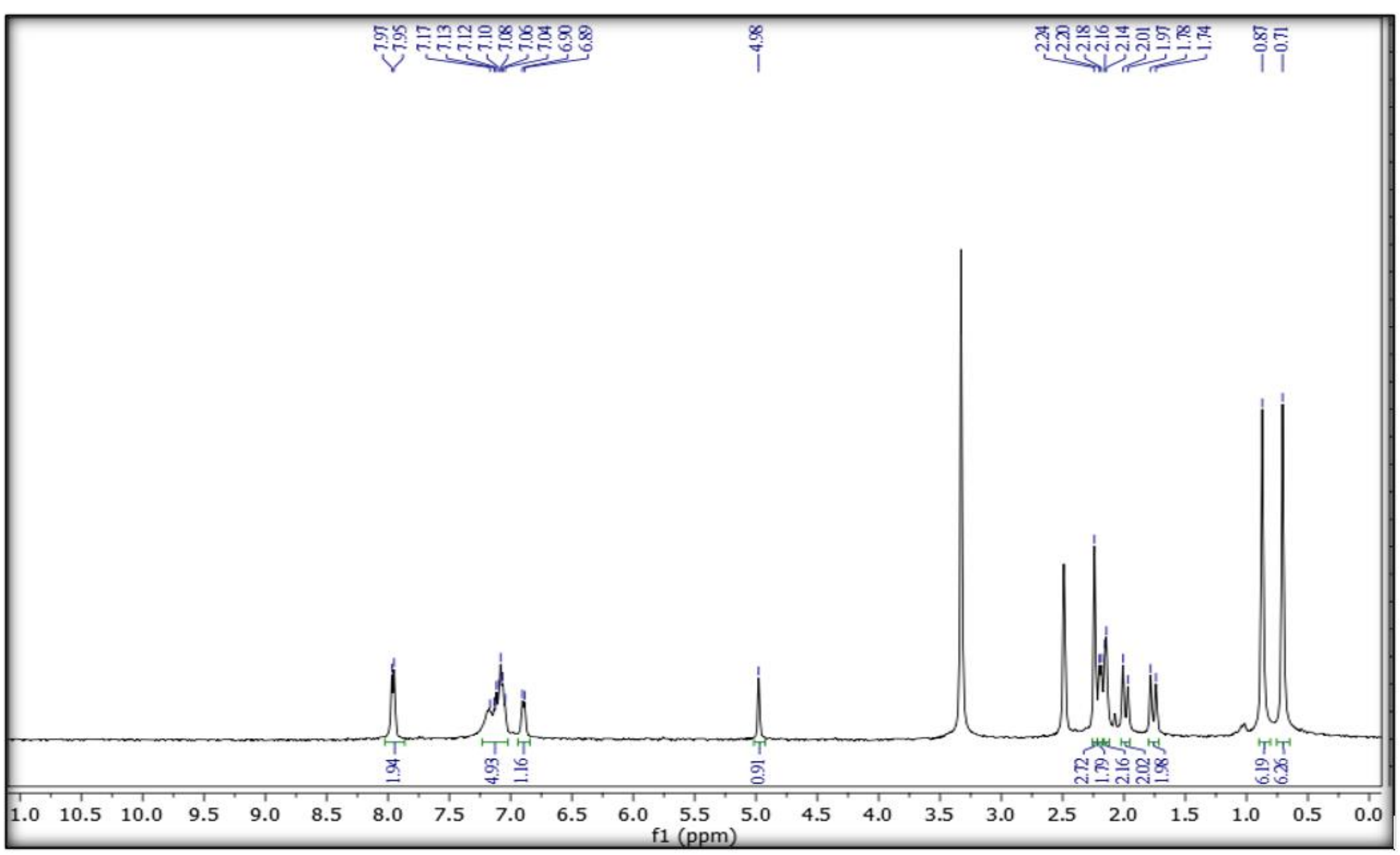

^{1}H NMR of **4i**

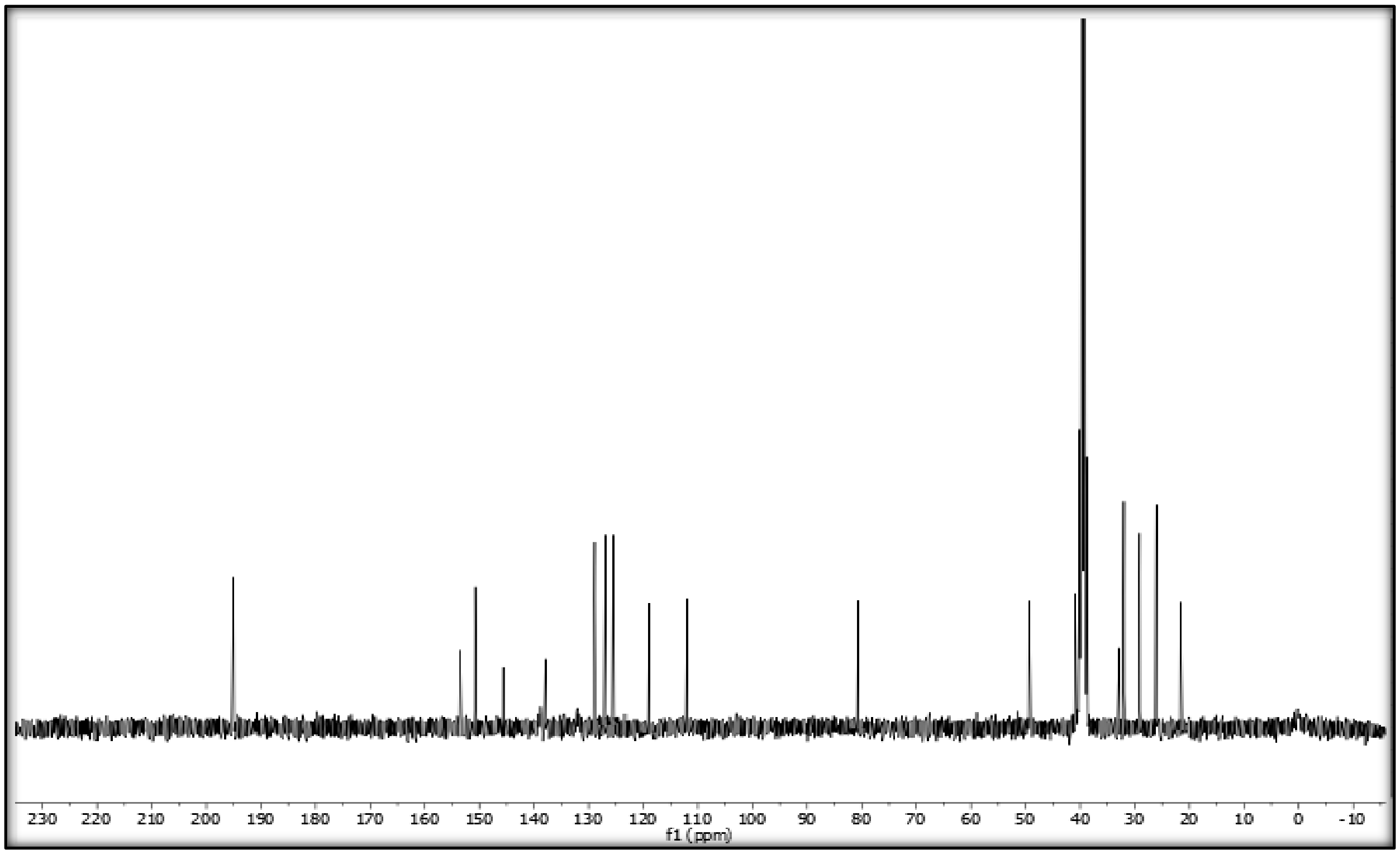

^{13}C NMR of **4i**

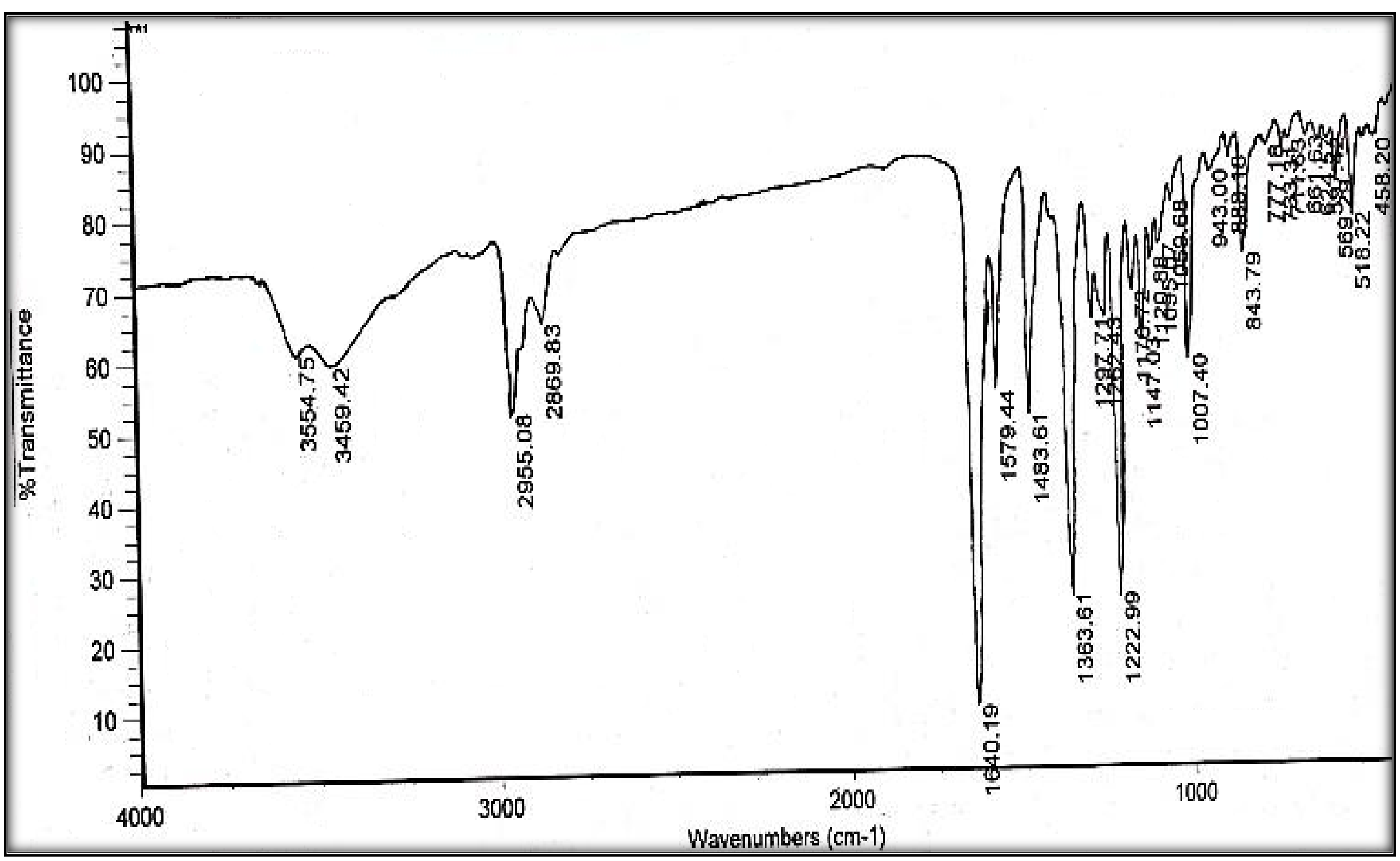

FT-IR of **4j**

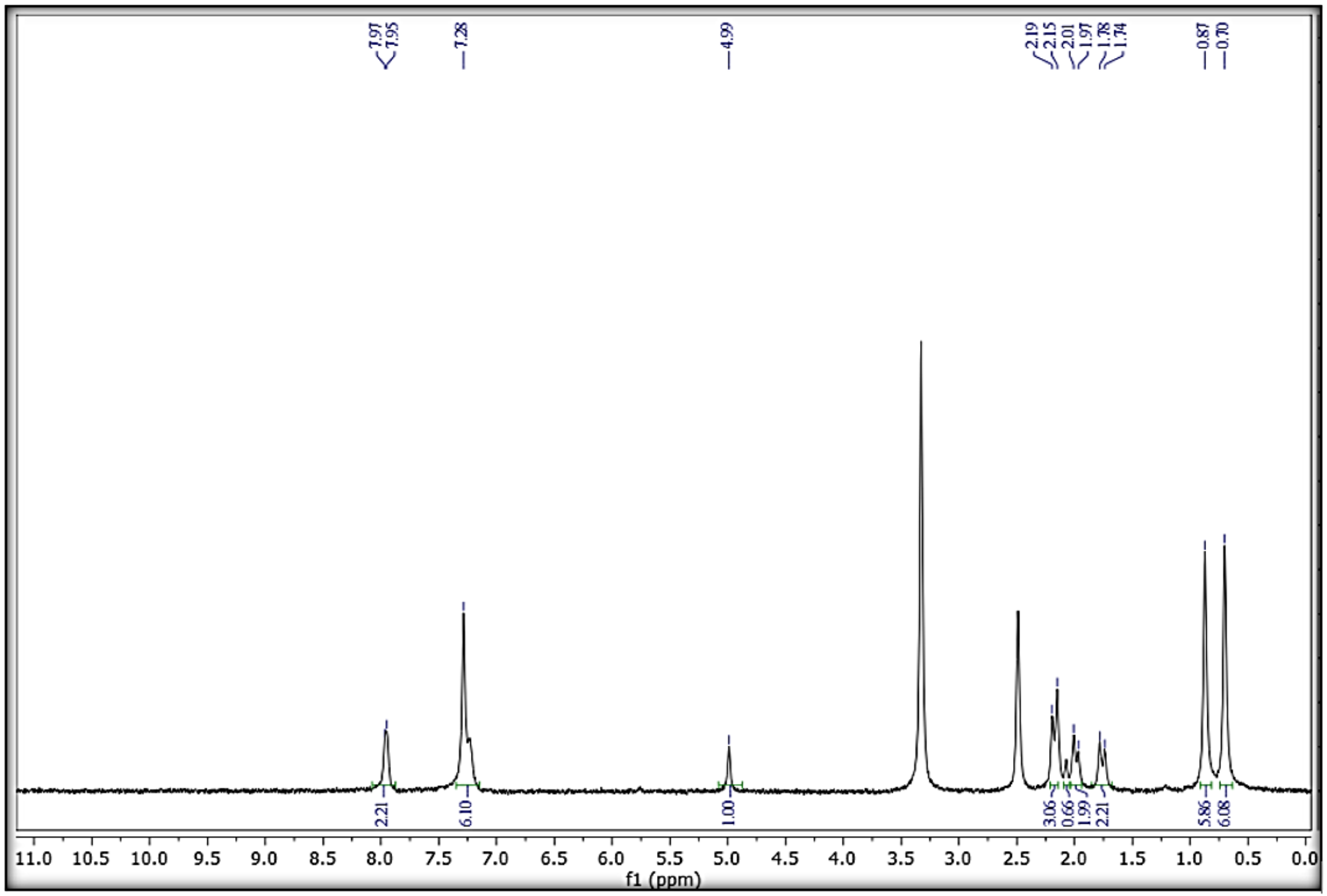
7.97
7.95
7.28
4.99
2.19
2.15
2.01
1.97
1.78
1.74
0.87
0.70
2.21
6.10
1.00
3.06
0.66
1.99
2.21
5.86
6.08
11.0 10.5 10.0 9.5 9.0 8.5 8.0 7.5 7.0 6.5 6.0 5.5 5.0 4.5 4.0 3.5 3.0 2.5 2.0 1.5 1.0 0.5 0.0
f1 (ppm)

^{1}H NMR of **4j**
